# Global gene expression profiling related to temperature-sensitive growth abnormalities in interspecific crosses between tetraploid wheat and *Aegilops tauschii*

**DOI:** 10.1371/journal.pone.0176497

**Published:** 2017-05-02

**Authors:** Ryusuke Matsuda, Julio Cesar Masaru Iehisa, Kouhei Sakaguchi, Ryoko Ohno, Kentaro Yoshida, Shigeo Takumi

**Affiliations:** 1 Laboratory of Plant Genetics, Graduate School of Agricultural Science, Kobe University, Kobe, Japan; 2 Departmento de Biotecnología, Facultad de Ciencias Químicas, Universidad Nacional de Asunción, San Lorenzo, Paraguay; 3 Graduate School of Science, Technology and Innovation, Kobe University, Kobe, Japan; Montana State University Bozeman, UNITED STATES

## Abstract

Triploid wheat hybrids between tetraploid wheat and *Aegilops tauschii* sometimes show abnormal growth phenotypes, and the growth abnormalities inhibit generation of wheat synthetic hexaploids. In type II necrosis, one of the growth abnormalities, necrotic cell death accompanied by marked growth repression occurs only under low temperature conditions. At normal temperature, the type II necrosis lines show grass-clump dwarfism with no necrotic symptoms, excess tillers, severe dwarfism and delayed flowering. Here, we report comparative expression analyses to elucidate the molecular mechanisms of the temperature-dependent phenotypic plasticity in the triploid wheat hybrids. We compared gene and small RNA expression profiles in crown tissues to characterize the temperature-dependent phenotypic plasticity. No up-regulation of defense-related genes was observed under the normal temperature, and down-regulation of wheat *APETALA1*-like MADS-box genes, considered to act as flowering promoters, was found in the grass-clump dwarf lines. Some microRNAs, including miR156, were up-regulated, whereas the levels of transcripts of the miR156 target genes *SPL*s, known to inhibit tiller and branch number, were reduced in crown tissues of the grass-clump dwarf lines at the normal temperature. Unusual expression of the miR156/*SPL*s module could explain the grass-clump dwarf phenotype. Dramatic alteration of gene expression profiles, including miRNA levels, in crown tissues is associated with the temperature-dependent phenotypic plasticity in type II necrosis/grass-clump dwarf wheat hybrids.

## Introduction

Allopolyploid speciation, which involves the full duplication of a hybrid genome [1], can be established through various processes, including interspecific hybrid formation, endoreduplication in hybrid plants, alteration of gene expression profiles, and genome stabilization [2, 3]. For successful allopolyploid speciation, normal growth and fertility of interspecific hybrids are essential. However, hybrid plants frequently fail to produce the next generation due to lethality and sterility. The molecular nature of the causative genes to hybrid lethality has recently been elucidated in *Arabidopsis* and other plant species [4]. A disease resistance (*R*) gene encoding the nucleotide binding leucine rich repeat (NB-LRR)-type protein is necessary to trigger hybrid necrosis in some intraspecific crosses of *Arabidopsis thaliana* [5,6]. One of the causal genes for the hybrid necrosis encodes SRF3 (Strubbelig Receptor Kinase 3), a receptor-like protein kinase, and complementarily interacts with the NB-LRR-type *R* gene located within the *RPP1* cluster [7]. In an interspecific cross of lettuce, *RIN4*, which encodes a protein interacting with multiple *R* gene products, is one of the causal genes of hybrid necrosis [8]. Therefore, it is postulated that hybrid necrosis is an autoimmune-like response induced by epistatically interaction of particular alleles of the *R* gene with particular alleles of genes elsewhere in the genome [4].

For the birth of common wheat (*Triticum aestivum* L., genome constitution AABBDD), normal triploid hybrids with an ABD genome were produced from an interspecific cross between tetraploid wheat (*Triticum turgidum* L., AABB genome) and diploid wild wheat *Aegilops tauschii* Coss. (DD genome) [9]. The evolutionary process generating common wheat can be artificially reproduced, and a number of synthetic hexaploid wheat plants have been produced from ABD hybrids of tetraploid wheat and *Ae*. *tauschii* [10–12]. On the other hand, triploid hybrids with tetraploid wheat sometimes show abnormal growth phenotypes such as germination failure, hybrid necrosis and hybrid sterility [13]. In particular, the following four types of the abnormal growth phenotypes are known in ABD hybrids between the tetraploid wheat cultivar Langdon (Ldn) and *Ae*. *tauschii* accessions: two types of hybrid necrosis (type II and type III), hybrid chlorosis, and severe growth abortion [14]. The hybrid incompatibilities in ABD triploids occur due to epistatic interaction between the AB and D genomes, which is postulated to depend on a phenomenon described by a Dobzhansky-Muller (DM) model [15]. The growth abnormalities in the triploid hybrids could act as postzygotic hybridization barriers between tetraploid wheat and *Ae*. *tauschii*, preventing the production of synthetic hexaploid wheat lines. Based on cytological and transcriptome analyses, an autoimmune response-like reaction may be associated with necrotic cell death in both types of hybrid necrosis in the ABD triploids [14,16].

Type II necrosis plants exhibit a distinct phenotype from those showing type III necrosis. Cell death occurs gradually from older to younger tissues in the hybrid lines showing type III necrosis, similarly to type I and type IV necrosis observed in other cross combinations of wheat [17], whereas a necrotic phenotype of type II necrosis lines is induced by low temperature (LT) conditions [14]. In addition, in the type II necrosis lines, stem elongation is markedly inhibited, and new leaf expansion is delayed under LT [16]. The type II necrosis lines exhibit a significant decrease in expression of genes related to the cell cycle and cell division occurs at the crown tissues including the shoot apical meristem [14]. Interestingly, the ABD triploids with type II necrosis show dramatic increase in tiller number and marked reduction of plant height at normal temperature [14,18]. No necrotic symptoms are observed and the start of senescence seemed to be delayed in the type II necrosis lines under normal temperature, and heading and flowering are remarkably delayed (by more than 3 weeks) in the type II necrosis plants compared with those in other synthetics with a normal phenotype under glasshouse conditions [18]. This characteristic under normal temperature corresponded to the so-called grass-clump dwarf phenotype, which has sometimes been observed in intra- and interspecific crosses of wheat and its relatives [19,20]. Complementary genes located on the AB and D genomes, named *Net1* and *Net2*, respectively, have been assumed to control type II necrosis, and *Net2* was assigned to the short arm of chromosome 2D [16]. Thus, epistatic interaction of *Net1* and *Net2* at the crown tissues may induce temperature-dependent phenotypic plasticity in the ABD triploids.

MicroRNAs (miRNAs) have been defined as a highly conserved class of small non-coding RNA molecules acting in post-transcriptional gene repression [21,22]. An RNA-induced silencing complex including miRNA directs decreased levels of the target mRNAs, which contain the complementary sequence, usually by mRNA cleavage in higher plants. In wheat and its relatives, many miRNAs are responsive to abiotic stress such as dehydration [23–26]. Mutations occurring in miRNA loci or in the miRNA-targeted sites sometimes result in developmental abnormalities in higher plants [27,28]. Overexpression of miR156 results in the extremely bushy dwarf phenotype of maize *Corngrass1* (*Cg1*) mutants [29], and the *Cg1* phenotype strongly resembles the grass-clump dwarf phenotype of wheat hybrids [18]. However, the relationship between grass-clump dwarfism and expression profiles of wheat miRNAs has never been studied.

Expression levels and patterns of small RNAs, including miRNAs and small interfering RNAs (siRNAs) and *trans*-acting siRNAs, could be altered in interspecific hybrids and allopolyploids [30]. The expression levels of small RNAs are negatively correlated with their target mRNA levels. Changes in small RNA levels have been observed in both intraspecific and interspecific F_1_ hybrids of rice, tomato and *Arabidopsis* [31–33]. In intersubspecies hybrids of rice, more small RNAs are down-regulated than up-regulated [32]. *Arabidopsis* miR163, which has expression levels correlated with levels of acetylation at H3K9 and trimethylation at H3K4, is highly expressed in *Arabidopsis thaliana*, repressed in *Arabidopsis arenosa*, and down-regulated in their synthetic allotetraploids [31,34]. Non-additive expression of miRNAs and *trans*-acting-siRNAs in allopolyploids may lead to morphological variation between *Arabidopsis* species [30]. Thus, small RNAs could contribute to phenotypic alteration in interspecific hybrids and allopolyploids. During wheat hybrid formation and allopolyploidization, the number of siRNAs corresponding to transposable elements greatly decreased [35]. The reduction in siRNAs could contribute to allopolyploid genome destabilization. In newly synthesized wheat allohexaploids, expression of miRNAs was greatly altered compared with the parental miRNA levels, possibly resulting in modified expression profiles of their target genes [36]. However, there are no reports of direct association of small RNAs with abnormal growth phenotypes in interspecific hybrids of wheat relatives.

One research objective of the present study was elucidation of the molecular mechanisms behind the temperature-dependent phenotypic plasticity in wheat ABD triploids with abnormal growth. Here, we report mRNA and miRNA transcriptome analyses in type II necrosis lines under distinct growth temperatures, and discuss association of miR156 with the grass-clump dwarf phenotype in the ABD triploids.

## Materials and methods

### Plant materials

In our previous study, a tetraploid wheat (*Triticum turgidum* subsp. *durum* Husn.) cultivar, Langdon (Ldn), was used as the female parent, and crossed with 122 *Aegilops tauschii* accessions to artificially produce triploid wheat hybrids [14]. Selfed seeds (F_2_ generation), called synthetic wheat, from the triploid F_1_ hybrids were obtained through unreduced gamete formation [10]. In this study, we used six synthetic hexaploid wheat lines (F_4_ generation), five derived from cross combinations between Ldn and five *Ae*. *tauschii* accessions, KU-2012, KU-2025, KU-2059, KU-2075 and KU-2159 [14], and one between a tetraploid wheat (*T*. *turgidum* subsp. *carthlicum* Á.Löve & D.Löve) accession, KU-138, and *Ae*. *tauschii* accession KU-2025 [18]. Three synthetic lines, Ldn/KU-2059, Ldn/KU-2075 and Ldn/KU-2159, showed a normal wild-type (WT) growth phenotype, whereas the Ldn/KU-2012, Ldn/KU-2025 and KU-138/KU-2025 (crt/KU-2025) synthetic wheat lines exhibited type II necrosis under LT. An F_2_ mapping population was previously generated by a cross between WT (Ldn/KU-2075) and type II necrosis (Ldn/KU-2025) synthetic hexaploids [16]. In the present study, some progeny from the F_2_ individuals were used for gene expression analyses. The *Net2*-homozygous plants exhibited a severe growth defect, and generally died in the winter season [16]. Phenotype of the *net2*-homozygous plants was normal, whereas the *Net2*-heterozygous plants frequently showed an intermediate phenotype of defective and delayed growth.

### mRNA transcriptome analysis

For microarray analysis, total RNA was extracted using an RNeasy Plant Mini kit (Qiagen, Hilden, Germany) from crown tissues of WT and type II necrosis lines grown at 24°C (normal temperature) under long day (18-h light and 6-h dark) conditions for 8 weeks. A KOMUGI 38k oligonucleotide DNA microarray (Agilent Technologies, Santa Clara, CA, USA) was supplied by the National BioResource Project-Wheat, Japan (https://www.nbrp.jp) for analysis, and the 38k microarray information was found in the Gene Expression Omnibus (GEO) database of the National Center for Biotechnology Information (NCBI) website under platform GPL9805 [37]. Hybridization of Cy3-labeled cRNA, washing and image scanning were performed according to our previous report [38]. Scanned images were analyzed using Feature Extraction Software with default parameters to obtain background-subtracted and spatially detrended processed signal intensities. After trimmed means excluding the 2% highest and lowest values were determined, the processed signal intensities of each probe were multiplied by the scaling factor (2500 divided by trimmed mean value) for normalization. Two independent experiments were conducted for each sample. All microarray data were deposited as GSE78784 in the NCBI GEO database (http://www.ncbi.nlm.nih.gov/geo/), including supplementary files, GSM2076765-2076770. The microarray data of the crown tissues under LT was used from our previous study in which total RNA was extracted from WT (Ldn/KU-2059) and type II necrosis (Ldn/KU-2025) lines exposed to 4°C for 6 weeks after grown at 24°C for 3 weeks [16]. The data of the LT-treated samples deposited as GSE24566 in the NCBI GEO database were here compared with those grown at the normal temperature.

BLASTx searches (*E* value < 1e^-10^) against the NCBI non-redundant protein database were conducted to predict the functions of probes and genes. The National BioResource Project’s KOMUGI website (http://www.shigen.nig.ac.jp/wheat/komugi/array/index.jsp) was also used to identify functions of the proteins partially encoded by the probes. Gene Ontology (GO) annotation was performed using Blast2GO [39]. Annotations were further refined using ANNEX [40] and mapped to plant-specific GO-Slim terms, both available in Blast2GO. GO terms of differentially expressed (mean of fold changes ≥ 3 or ≤ 1/3, subjected to Student’s *t*-test with Benjamini and Hochberg multiple testing correction at a false discovery rate [FDR] of 5%) probes were extracted and compared. Based on the GO term enrichment analysis (Fisher’s exact test, FDR < 0.05), the differentially encountered probes were input for biological processes (level 3).

Heat map analysis of the selected probes was conducted using Multiple Experiment Viewer version 4.8.1 [41]. Pearson correlation was estimated based on the log2 fold change values of the probes, and hierarchical clustering by average linkage was performed using the correlation values as distance metrics.

### Deep sequencing of small RNAs

For the first screening of miRNA associated with the grass-clump dwarfism via small RNA sequencing, total RNA was extracted from crown tissues of WT and type II necrosis lines grown at normal temperature and 4°C under long-day (18-h light and 6-h dark) conditions for 8 weeks using Sepasol-RNA I Super G solution (Nacalai Tesque, Kyoto, Japan). For each sample, crown tissues of at least two independent plants were bulked with no biological replications. Small RNA libraries were prepared using a TruSeq Small RNA Library Preparation Kit (Illumina, San Diego, CA, USA) and RNA 3’ adapter and RNA 5’ adapter were respectively added using truncated T4 RNA ligase 2 (New England BioLabs, Ipswich, MA, USA) and T4 RNA ligase 1 (New England BioLabs). cDNA was synthetized using the 3’ adapter-recognizing primer, and after PCR, products of around 150 bp were selected. Single-end sequencing was performed with a TruSeq SBS Kit v3-HS (Illumina) on a HiSeq2000 platform (Illumina) according to the manufacturer’s instructions. Files containing raw sequence data were deposited in the sequence read archive of DDBJ (accession number DRA004554).

### Extraction and annotation of miRNAs

From the raw sequence reads, stop oligonucleotides, binding to the 3' adapter to prevent further ligation, were removed and then adapter sequences were trimmed (S1 Fig). The trimmed reads were BLASTn searched against Rfam 11.0 [42] to remove reads with homology ≥ 90% (plus/plus strand search, without low-complexity masking) to non-coding RNAs such as tRNAs, rRNAs and snoRNAs. Of the remaining reads, those with length ≥ 18 bp and ≤ 30 bp were aligned to the repeat-masked A- and D-genome sequences [43,44] using Bowtie version 1.0.0 [45], reporting all valid alignments with a perfect match (-v 0 -a). Rfam and Repbase 18.10 databases [46] were used for masking of the A- and D-genomes with RepeatMasker version open-4.0.1 (http://www.repeatmasker.org).

The aligned data were used to predict novel miRNAs with mireap version 0.2 (http://sourceforge.net/projects/mireap) with the following parameters: minimal miRNA length of 18 bp and maximal of 30 bp, minimal reference miRNA length of 18 bp and maximal of 26 bp, maximal space between miRNA and miRNA* of 400 bp, maximal bulge of miRNA and miRNA* of 3 bp, flank sequence length of miRNA precursor of 20 bp and maximal free energy allowed for a miRNA precursor of -20. The obtained miRNAs were BLASTn searched against the miRBase 19.0 plant miRNA database [47] (homology ≥ 90%, plus/plus strand search, without low-complexity masking) to separate known miRNAs from the novel miRNAs. Reads that were unaligned to genomic sequence that remained after mireap analysis were also BLASTn searched using the same parameters against the miRBase to rescue the known miRNAs.

### miRNA target prediction

For miRNA target prediction, psRNATarget (2017 Update) [48] were used with scoring schema V2 (2017 release) against *Triticum aestivum*, unigene, DFCI gene index (TAGI) version 12 and *Triticum aestivum* cDNA, Ensembl Plants, release-31. Targets with maximum expectation values > 2 were discarded. Unannotated genes were BLASTx searched against the NCBI non-redundant protein database (*E*-value < 10^−3^).

### Quantitative RT-PCR (qRT-PCR)

For qRT-PCR, each plant was grown at 23°C for 3 weeks and total RNA was extracted from the crown tissues using Sepasol-RNA I (Nacalai Tesque, Kyoto, Japan). Crown tissues of at least two independent plants were bulked for each synthetic hexaploid line. First-strand cDNA was synthesized from DNase I-treated RNA samples using ReverTra Ace Reverse Transcriptase (Toyobo, Osaka, Japan) and an oligo(dT)_20_ primer. The accumulation of each gene transcript was detected by qRT-PCR using a LightCycler 480 Real-Time PCR System (Roche Diagnostics, Mannheim, Germany) with the gene-specific primer sets listed in S1 Table. These primers amplify genes expressed from the A-, B- and D-genomes. The *Actin* gene was used as an internal control. The rate of amplification was monitored using THUNDERBIRD SYBR qPCR mix (Toyobo, Osaka, Japan) according to the manufacturer’s protocol. The relative expression level was calculated as 2^-ΔΔCt^ of three technical replicates [49], where ΔCt is the difference in number of PCR cycles required to reach the log phase of amplification of the target gene relative to *Actin*.

For validation of the first screening of miRNA assocaited with grass-clump dwarfism via small RNA sequencing, we conducted qRT-PCR of miRNAs. Total RNA was extracted from crown tissues of the synthetic hexaploid lines and F_2_ individuals of the Ldn/KU-2075//Ldn/KU-2025 population. Bulked RNA samples of each *Net2* genotype were extracted from the crown tissues of five F_2_ individuals. The accumulation of each miRNA was detected by qRT-PCR using the first-strand cDNA samples synthesized by a Mir-X miRNA First-Strand Synthesis Kit (BD Biosciences Clontech, Tokyo, Japan). qRT-PCR was conducted using SYBR Advantage premix (BD Biosciences Clontech) with a miRNA-specific primer and mRQ3’ adapter primer. The miRNA-specific primers for qRT-PCR are listed in S2 Table. 18S rRNA was used as an internal control. The relative expression level was calculated as 2^-ΔΔCt^ of three technical replicates.

### Phylogenetic analysis

The cDNA sequences of *SQUAMOSA PROMOTER BINDING PROTEIN-LIKE* (*SPL*) and MADS-box genes were based on recent reports [50–52], and the nucleotide sequences were obtained from public DNA databases of rice (TIGR, http://rice.tigr.org/), *Arabidopsis* (TAIR, http://www.arabidopsis.org), barley and wheat (NCBI, http://www.ncbi.nlm.nih.gov), and the deduced amino acid sequences were predicted by ORF Finder (http://www.ncbi.nlm.nih.gov/projects/gorf/gorf.html). Conserved domains of the amino acids were confirmed using InterProScan (http://www.ebi.ac.uk/Tools/pfa/iprscan/). Multiple sequence alignments of the amino acid sequences were carried out using the ClustalW computer program (gap penalty = 8, gap extension penalty = 2, PAM protein weight matrix) of MEGA ver. 5.05 software [53], and a phylogenetic tree was constructed by the maximum likelihood (Poisson model of amino acid substitution and 4–γcategory model) and neighbor-joining (bootstrap = 1000) methods.

### 5’-RACE analysis of wheat *SPL* transcripts

Based on the wheat *SPL* sequences, gene-specific primers were designed for determining the 5’ ends of the wheat *SPL* cDNAs, and rapid amplification of cDNA ends (RACE)-PCR was conducted using a BD SMART RACE cDNA Amplification Kit (BD Biosciences Clontech). Each amplified PCR product was cloned into vector pMD20-T using Mighty TA-cloning Reagent Set for PrimeSTAR (Takara Bio). The nucleotide sequences of the 5’-RACE PCR products were determined using a Big Dye terminator cycle sequencing kit (Applied Biosystems, Foster City, CA, USA) and an Applied Biosystems 3730*xl* DNA Analyzer. The nucleotide sequences were analyzed using GENETYX-MAC version 12.00 software (Whitehead Institute for Biomedical Research, Cambridge, MA, USA).

## Results

### Microarray analysis of gene expression profiles in type II necrosis lines

**Fig 1:**
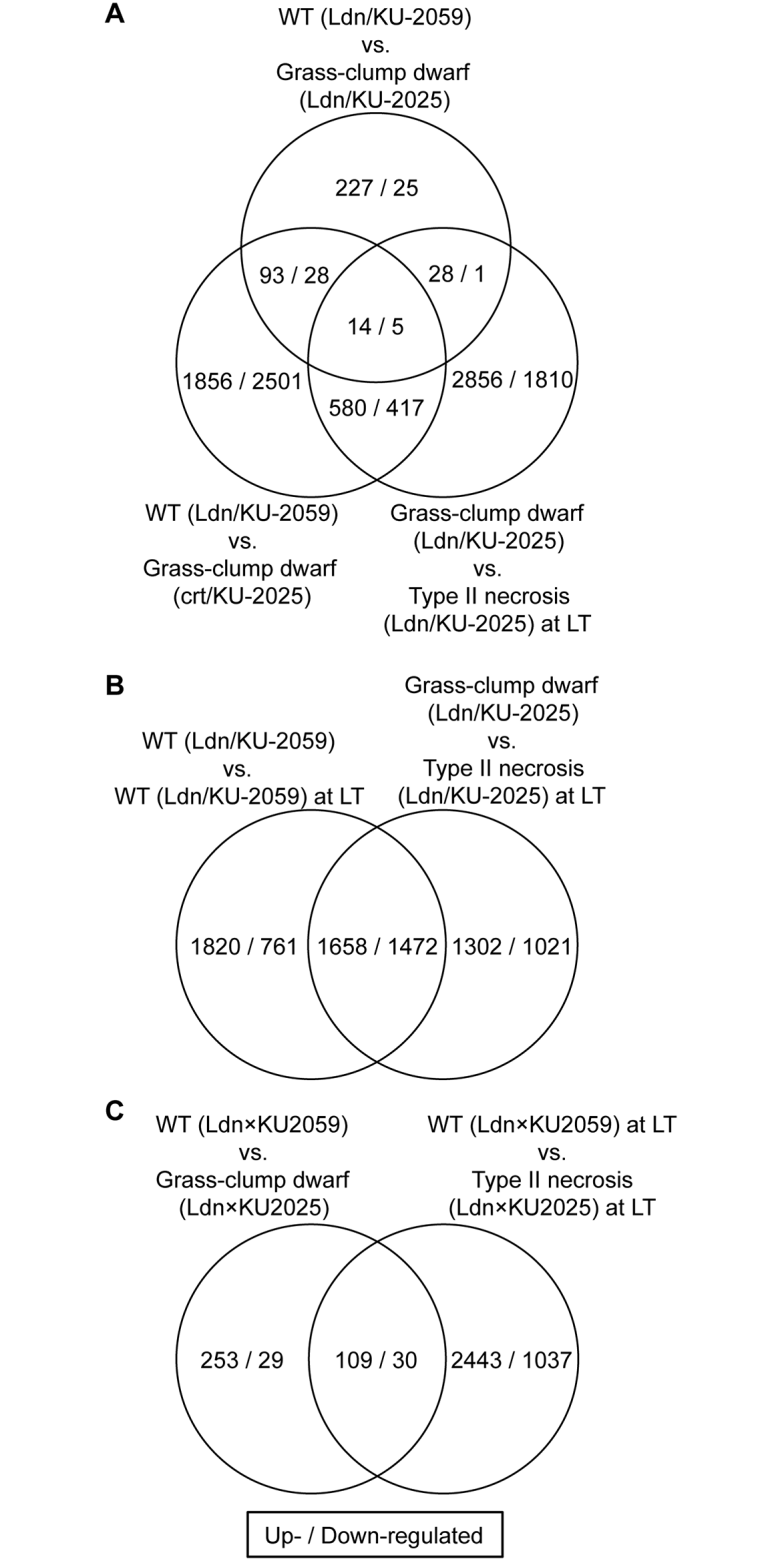
Comparison of microarray data for the examined crown tissues. (A) Venn diagram of differentially expressed up- and down-regulated genes in three examined lines: WT (Ldn/KU-2059) and grass-clump dwarf (Ldn/KU-2025 and crt/KU-2025). (B) Venn diagram of differentially expressed up- and down-regulated genes in the crown tissues of Ldn/KU-2059 and Ldn/KU-2025 under LT. (C) Venn diagram of differentially expressed up- and down-regulated genes in Ldn/KU-2025 under the normal temperature and LT.

To comprehensively compare gene expression profiles between WT (Ldn/KU-2059) and two type II necrosis lines (Ldn/KU-2025 and crt/KU-2025) under normal temperature, we analyzed their transcriptomes using a wheat-specific 38k oligo DNA microarray [37]. After hybridization with the RNA samples, probes showing at least a threefold difference (FDR<0.05) in signal intensity compared to the WT were defined as either up- or down-regulated genes. Of the 37,826 probes on the wheat microarray, 362 (0.96%) probes were regarded as reflecting genes up-regulated in crown tissues of the Ldn/KU-2025 line compared with WT plants, and 59 (0.16%) were regarded as down-regulated. Similarly, 2,543 (6.72%) up-regulated and 2,591 (6.85%) down-regulated probes were observed in crown tissues of the crt/KU-2025 line compared with WT plants. Of them, 107 were commonly up-regulated between both the Ldn/KU-2025 and crt/KU-2025 lines compared with WT plants, and 33 were down-regulated (Fig 1A). Differences in signal intensity of all 37,826 probes relative to WT were compared between the Ldn/KU-2025 and crt/KU-2025 lines, and a significant positive correlation (*R* = 0.651, *P*<0.001) was detected (Table 1).

**Table 1:** Comparison of gene expression profiles between crown tissues of type II necrosis-expressing and grass-clump dwarf plants of synthetic hexaploid wheat.

Query	Target expression profile	Correlation coefficient
in Ldn/KU-2025 vs. Ldn/KU-2059	crt/KU-2025 vs. Ldn/KU-2059	0.651***
in Ldn/KU-2025 vs. Ldn/KU-2059	Ldn/KU-2025 vs. crt/KU-2025	0.301**
in crt/KU-2025 vs. Ldn/KU-2059	Ldn/KU-2025 vs. crt/KU-2025	0.970***
in Ldn/KU-2025 vs. Ldn/KU-2025-LT^a^	Ldn/KU-2059 vs. Ldn/KU-2059-LT^a^	0.887***
in Ldn/KU-2025 vs. Ldn/KU-2025-LT	Ldn/KU-2025-LT vs. Ldn/KU-2059-LT	0.507***
in Ldn/KU-2025 vs. Ldn/KU-2025-LT	Ldn/KU-2025 vs. Ldn/KU-2059	-0.074***
in Ldn/KU-2059 vs. Ldn/KU-2059-LT	Ldn/KU-2025-LT vs. Ldn/KU-2059-LT	-0.541***
in Ldn/KU-2059 vs. Ldn/KU-2059-LT	Ldn/KU-2025 vs. Ldn/KU-2059	0.331***
in Ldn/KU-2025-LT vs. Ldn/KU-2059-LT	Ldn/KU-2025 vs. Ldn/KU-2059	0.553***

The Pearson coefficient values were calculated based on the differences in signal intensities of seven sets of all 37,826 probes between the wild-type and abnormal growth hybrids.

Significant correlations:

**, *P*<0.01;

***, *P*<0.001.

^a^Mizuno et al. [16]

**Fig 2:**
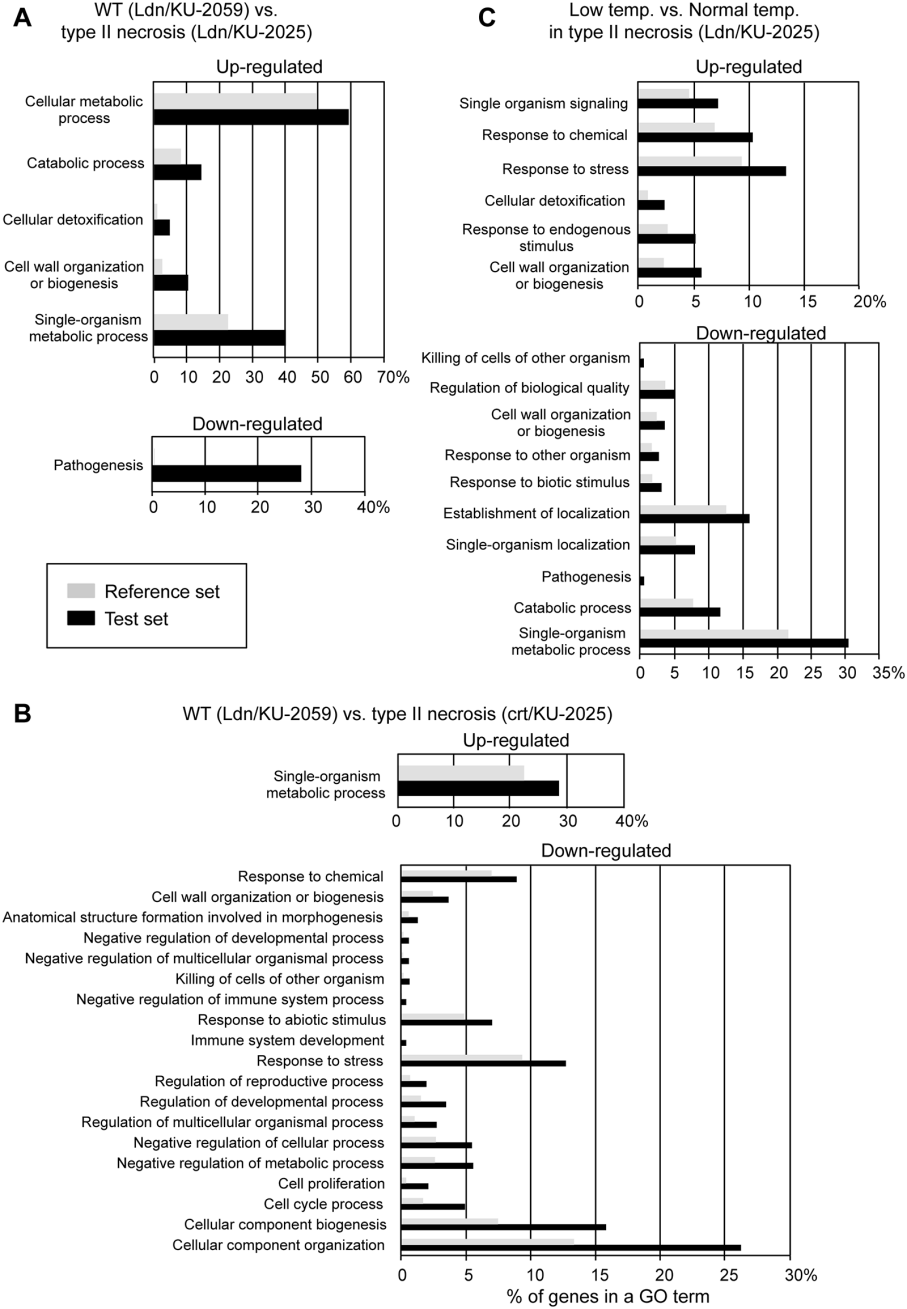
GO terms enriched among differentially expressed genes. Black bars indicate percentages of differentially encountered probes by microarray analysis (test set), and gray bars represent percentages of probes on the used array (reference set). (A, B) Comparison of transcriptomes between type II necrosis and WT synthetic wheat lines. Up- and down-regulated genes in the type II necrosis line Ldn/KU-2025 (A) and the type II necrosis line crt/KU-2025 (B) relative to the WT line (Ldn/KU-2059) were categorized into some biological groups. (C) Comparison of transcriptomes between normal temperature and LT in type II necrosis. Up- and down-regulated genes in the type II necrosis line Ldn/KU-2025 under LT relative to the necrosis line at normal temperature were categorized into some biological groups. The GO term enrichment analysis was performed using Fisher’s exact test with FDR < 0.05, the differentially encountered probes were categorized into biological processes (level 3).

**Fig 3:**
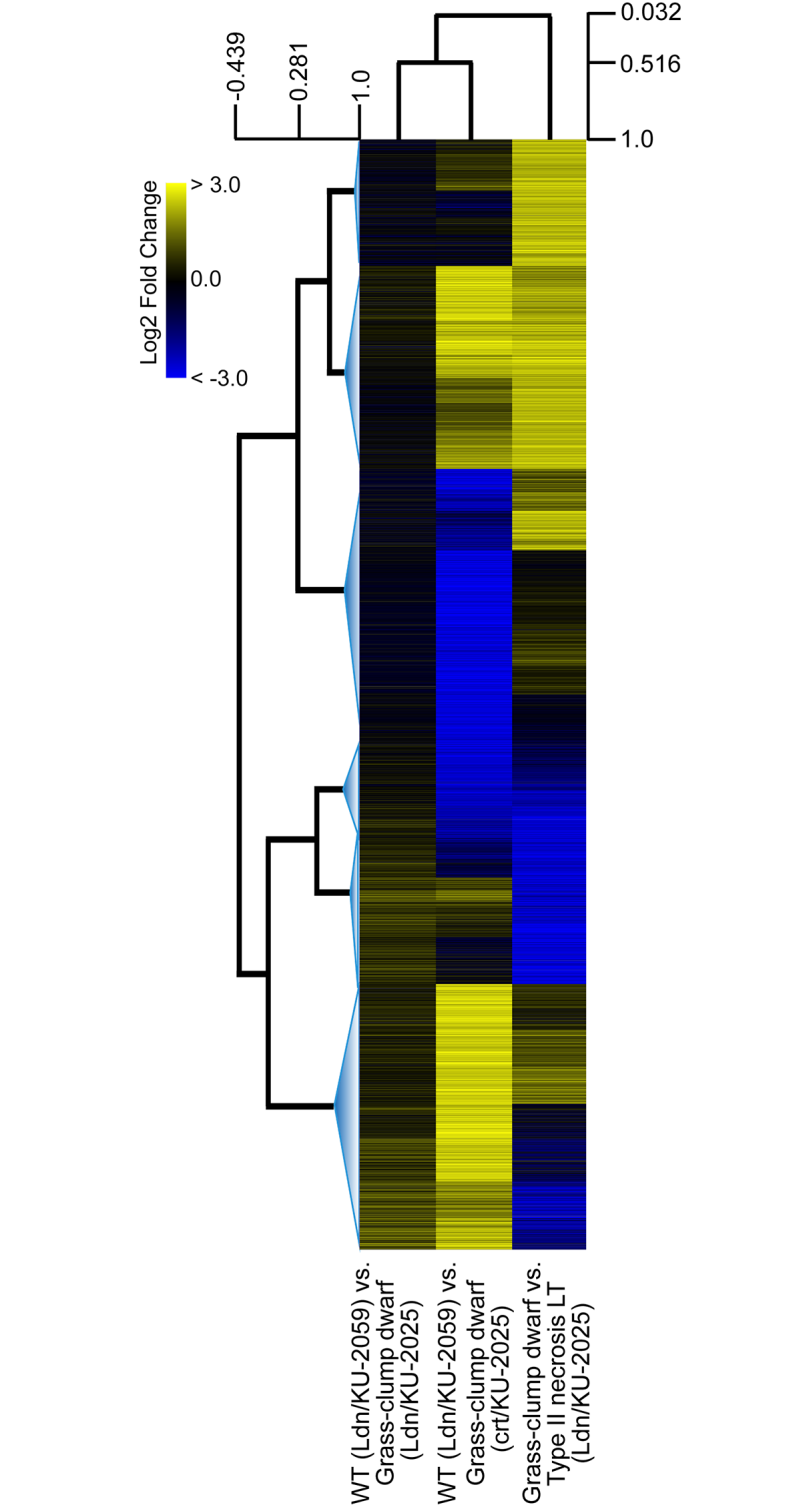
Heat map for the altered gene expression levels of the selected 9,974 probes in the three examined crown tissues; WT (Ldn/KU-2059) and grass-clump dwarf (Ldn/KU-2025), WT (Ldn/KU-2059) and grass-clump dwarf (Ldn/KU-2025), and grass-clump dwarf (Ldn/KU-2025) under the normal temperature and LT. The branches of the trees are arranged according to the Pearson correlation coefficients. The distance threshold adjustment resulted in six major clusters. The colour-scale shows log_2_ fold change values.

Based on homology searches of the wheat EST database with probe sequences, the up- and down-regulated probes were analyzed by the GO term enrichment analysis, and categorized into some biological process groups (Fig 2). Of the up-regulated probes, single-organism metabolic process-related genes were the most frequently encountered under normal temperature in the Ldn/KU-2025 (59.0%) and crt/KU-2025 (28.3%) lines, respectively. Genes related to cellular metabolic process-related genes were also abundantly expressed in the type II necrosis lines. In contrast, many more cell component organization-, cell component biogenesis- and response to stress-related genes were down-regulated in crown tissues of the crt/KU-2025 line than in the Ldn/KU-2025 line. Heat map analysis using the intensities of probes with the altered gene expression levels (in total 9,974 probes) revealed that much more enhanced change rates of the gene expression levels were observed in crt/KU-2025 than in Ldn/KU-2025 (Fig 3). Next, probes belonging to six functional categories (carbohydrate metabolism, lipid metabolism, energy, photosynthesis, transcription factor and defense response) were selected, and differences in the probe signal intensity relative to WT were compared between crown tissues of the two type II necrosis lines under normal temperature. Positive correlations of probe intensity differences in all examined categories were significant (*P*<0.001) between crown tissues of the Ldn/KU-2025 and crt/KU-2025 lines (S2 Fig). These observations suggest that gene expression profiles generally resemble each other for crown tissues of the two type II necrosis lines under normal temperature.

Among the probes up-regulated in Ldn/KU-2025 under normal temperature, defense-related genes such as peroxidase-encoding genes and photosynthesis-related genes including the genes for chlorophyll a/b binding protein and ribulose-1,5-bisphosphate carboxylase were found (S3 Table). In crt/KU-2025, many defense-related genes such as those for pathogenesis-related (PR) proteins and germin-like protein genes were up-regulated under normal temperature. The probes down-regulated in the crown tissues of Ldn/KU-2025 included many defense-related genes including the genes for NBS-LRR-type disease resistance protein, and defense-related genes such as defensin and some histone genes were dramatically repressed in the crown tissues of crt/KU-2025 (S3 Table). Several transcription factor genes, encoding WRKY, NAC and bZIP family proteins, were highly up-regulated in the crown tissues of crt/KU-2025 under normal temperature (Table 2), whereas some MADS-box transcription factor genes were remarkably down-regulated in the crown tissues of crt/KU-2025 (Table 3).

**Table 2:** Top 11 transcription factor genes identified by microarray analysis as up-regulated in crown tissues of the type II necrosis line (crt/KU-2025) under normal temperature compared with WT.

Probe	Accession no.	Protein	*E*-value	Log_2_ ratio
rwhcs14n21_490	EU665435	*Triticum aestivum* WRKY10 transcription factor	5.89E^-37^	5.1890
MUGEST2003_23lib_Contig5310_624	EF368363	*Triticum aestivum* WRKY transcription factor	4.82E^-97^	3.9827
wheat0130Contig2276	HQ858799	*Brachypodium distachyon* transcription factor MYB44-like	5.81E^-95^	3.6756
rwhh21m11	JN634080	*Zea mays* secondary wall NAC transcription factor 3	4.05E^-56^	3.5131
wheat0130Contig5738	DQ102384	*Hordeum vulgare* root abundant factor	2.34E^-20^	2.9430
wheat0130Contig5692	EU962117	*Zea mays* zinc finger family protein	1.46E^-35^	2.6656
wheat0130Contig6080	DQ863115	*Brachypodium distachyon* WRKY transcription factor 54	2.35E^-38^	2.6062
whgc20o07_524	EU974154	*Hordeum vulgare* heat stress transcription factor a-5-like	6.84E^-123^	2.5452
wheat0130Contig13733	EU956324	*Zea mays* bZIP transcription factor	4.88E^-72^	2.4133
wheat0130Contig12300	EF368365	*Triticum aestivum* WRKY13 transcription factor	3.85E^-146^	2.3603
MUGEST2003_23lib_Contig9158_829	EU958286	*Zea mays* GATA transcription factor 25	3.63E^-96^	2.1843

Probes used in the microarray analysis are referred to the NBRP KOMUGI website (http://www.shigen.nig.ac.jp/wheat/komugi/array/index.jsp).

**Table 3:** Top 13 transcription factor genes identified by microarray analysis as down-regulated in crown tissues of the type II necrosis line (crt/KU-2025) under normal temperature compared with WT.

Probe	Accession no.	Protein	*E*-value	Log_2_ ratio
rwhf1p23	DQ512331	*Triticum aestivum* MIKC-type MADS-box transcription factor WM7	9.02E^-41^	-8.6439
ncbi_gi_40644779_305	AM502879	*Triticum aestivum* MIKC-type MADS-box transcription factor WM13	8.02E^-125^	-4.8242
rwhfl24d04	DQ512346	*Elymus nutans* MIKC-type MADS-box transcription factor WM8	4.84E^-23^	-4.7839
rwhyf2e11	AB458519	*Triticum aestivum* AP2 transcription factor	6.37E^-64^	-4.1078
MUGEST2003_23lib_Contig10290_927	GU108423	*Hordeum vulgare* Ethylene-responsive transcription factor WIN1-like	8.63E^-94^	-4.0422
ATU17891	AF370307	*Arabidopsis thaliana* G-box binding factor 3	0	-3.3838
whec14a15_212	HQ858843	*Brachypodium distachyon* AP2-like ethylene-responsive transcription factor AIL5	3.95E^-84^	-2.8303
MUGEST2003_23lib_Contig1699_433	HQ858769	*Zea mays* RWP-RK domain-containing protein	1.21E^-99^	-2.6259
wheat0130Contig1725	AY280870	*Triticum aestivum* MADS-box transcription factor BM5A	3.99E^-35^	-2.4580
wheat0130Contig8821	DQ353856	*Triticum aestivum* homeodomain-leucine zipper transcription factor	5.85E^-27^	-2.4383
whcs5a15_376	AY685119	*Oryza sativa* AP2-like ethylene-responsive transcription factor BBM2-like	8.43E^-120^	-1.8943
ncbi_gi_11877790_965	EU965513	*Zea mays* transcription factor Dp-1	2.15E^-176^	-1.8615
ncbi_gi_469055_1362	D12919	*Triticum aestivum* transcription factor HBP-1a	3.22E^-132^	-1.6762

### Effect of growth temperature on gene expression profiles in type II necrosis

Correlation of the log_2_ ratios of the gene expression levels in the crown tissues was examined between the two growth temperature conditions based on the mRNA microarray data with 37,826 probes. The microarray data of the crown tissues under LT was used from our previous study [16] and compared with those under the normal temperature condition. Of the 37,826 probes on the wheat microarray, 2,960 (7.83%) probes were regarded as reflecting genes up-regulated in crown tissues of the WT line (Ldn/KU-2059) under LT, and 2,493 (6.59%) were regarded as down-regulated. Similarly, 3,478 (9.19%) up-regulated and 2,233 (5.90%) down-regulated probes were observed in crown tissues of the Ldn/KU-2025 line under LT (type II necrosis). Of them, 1,658 were commonly up-regulated between both the WT and Ldn/KU-2025 lines under LT, and 1,472 were down-regulated (Fig 1B). Significant positive correlations (*P*<0.001) of differences in signal intensity of the 37,826 probes under normal temperature relative to that under LT were observed between the WT synthetic line Ldn/KU-2059 and type II necrosis line Ldn/KU-2025 (Table 1). Out of the commonly up-regulated 107 probes in the two grass-clump dwarf lines, 14 (13.08%) were up-regulated by LT in the Ldn/KU-2025 line (Fig 1A). Five (15.15%) of the commonly down-regulated 33 probes were down-regulated by LT in Ldn/KU-2025. On the other hand, 362 (0.96%) probes were regarded as reflecting genes up-regulated between Ldn/KU-2059 and Ldn/KU-2025 under normal temperature, and 59 (0.16%) were regarded as down-regulated. 2,552 (6.75%) up-regulated and 1,067 (2.82%) down-regulated probes were observed between Ldn/KU-2059 and Ldn/KU-2025 under LT. Of them, 109 were commonly up-regulated between both the Ldn/KU-2059 and Ldn/KU-2025 lines under the two growth temperature conditions, and 30 were down-regulated (Fig 1C). Differences in the probe signal intensity between Ldn/KU-2059 and Ldn/KU-2025 under LT was positively (*P*<0.001) correlated with those under normal temperature. These observations indicated that the similar set of genes was transcriptionally altered by LT between WT and type II necrosis. On the other hand, differences in the probe signal intensity between the two growth conditions were negatively (*P*<0.001) correlated with those between Ldn/KU-2059 and Ldn/KU-2025. Thus, changes of gene expression levels via the growth temperatures were inconsistent with those via development of the growth abnormalities, and expression of some genes was specifically changed in the type II necrosis line in response to the growth temperatures.

The GO term enrichment analysis showed that response to stress and chemical-related genes were more up-regulated in the Ldn/KU-2025 line under normal temperature than under LT (Fig 2C). The heat map indicated that the LT-induced alteration of gene expression levels in Ldn/KU-2025 was distinct from the gene expression change accompanied with the difference of AB-genome donor under normal temperature (Fig 3). Of the down-regulated probes, single-organism metabolic process- and establishment of localization-related genes were the most frequently encountered under normal temperature in the Ldn/KU-2025. Among the probes up-regulated in Ldn/KU-2025 under LT, a lot of defense-related genes including the genes for chitinase and a PR protein were found (S3 Table). Some LT response-related and WRKY transcription factor genes were highly up-regulated in crown tissues of Ldn/KU-2025 under LT compared to normal temperature (Table 4). In Ldn/KU-2025 under LT, MADS-box and WRKY transcription factor genes were greatly down-regulated (Table 5).

**Table 4:** Top 13 transcription factor genes identified by microarray analysis as up-regulated in crown tissues of the type II necrosis line (Ldn/KU-2025) under LT relative to normal temperature.

Probe	Accession no.	Protein	*E*-value	Log_2_ ratio
ncbi_gi_32128580_908	GU902790	*Triticum monococcum* nuclear transcription factor Y subunit B9	2.11E^-123^	7.5085
ncbi_gi_17226800_612	AF376136	*Triticum aestivum* CRT/DRE-binding factor	1.31E^-129^	3.9827
wheat0130Contig8964	EF028775	*Triticum aestivum* CBFIVb-21.1	5.19E^-33^	3.9625
whfl_allContig1391	DQ512359	*Triticum aestivum* MIKC-type MADS-box transcription factor WM20	3.47E^-111^	3.7233
MUGEST2003_23lib_Contig5735_588	HQ858820	*Brachypodium distachyon* AP2-like ethylene-responsive transcription factor At1g16060	3.05E^-50^	2.5165
wheat0130Contig13733	EU956324	*Triticum aestivum* FD-like 15 protein	4.88E^-72^	2.1250
rwhdl26m07	EU955555	*Hordeum vulgare* zinc finger	2.09E^-23^	2.0580
MUGEST2003_23lib_Contig4256_1071	AM502900	*Triticum aestivum* MIKC-type MADS-box transcription factor WM30	1.17E^-125^	1.9967
wheat0130Contig8821	DQ353856	*Hordeum vulgare* DNA-binding expressed	5.85E^-27^	1.9880
wheat0130Contig6503	HQ738284	*Lophopyrum elongatum* stress related bZIP transcription factor	1.22E^-81^	1.9226
MUGEST2003_23lib_Contig9578_175	EU665440	*Triticum aestivum* WRKY45 transcription factor	1.02E^-09^	1.8408
wheat0130Contig13695	JN681191	*Triticum aestivum* ethylene responsive transcription factor 5a	1.17E^-99^	1.7146
whcs5m19_789	HQ858782	*Zea mays* FAR1 transcription factor	0	1.6973

**Table 5:** Top 15 transcription factor genes identified by microarray analysis as down-regulated in crown tissues of the type II necrosis line (Ldn/KU-2025) under LT relative to under normal temperature.

Probe	Accession no.	Protein	*E*-value	Log_2_ ratio
rwhfl48m16	DQ512331	*Triticum aestivum* MIKC-type MADS-box transcription factor WM7	4.50E^-39^	-7.7028
wheat0130Contig14238	DQ512346	*Triticum aestivum* MADS-box transcription factor TaAGL29	5.61E^-128^	-7.0979
MUGEST2003_23lib_Contig16395_223	DQ840409	*Brachypodium distachyon* WRKY transcription factor 17	8.57E^-36^	-5.7331
wheat0130Contig3983	DQ286566	*Triticum aestivum* WRKY transcription factor	2.56E^-86^	-5.0069
wheat0130Contig6325	EU665449	*Oryza sativa* WRKY transcription factor 48	2.20E^-35^	-4.9170
rwhr20m02	EF368364	*Triticum aestivum* WRKY53-b transcription factor	2.01E^-79^	-4.5632
wheat0130Contig10100	JX679079	*Triticum aestivum* WRKY transcription factor	5.98E^-38^	-4.3104
ATU17891	AF370307	*Arabidopsis thaliana* G-box binding factor 3	0	-4.2163
wheat0130Contig8019	EF397616	*Triticum aestivum* WRKY19-b transcription factor	6.22E^-30^	-4.1103
MUGEST2003_23lib_Contig8637_301	HQ858785	*Zea mays* MYB transcription factor	6.73E^-34^	-3.9817
ncbi_gi_32128574_754	DQ334412	*Triticum aestivum* ethylene-responsive transcription factor ERFL1a	5.26E^-48^	-3.8615
whcs5a15_376	AY685119	*Oryza sativa* AP2-like ethylene-responsive transcription factor BBM2-like	8.43E^-120^	-3.6171
MUGEST2003_23lib_Contig1326_155	DQ863106	*Hordeum vulgare* WRKY transcription factor 22	1.17E^-13^	-3.4158
MUGEST2003_23lib_Contig1699_433	HQ858769	*Zea mays* RWP-RK domain-containing protein	1.21E^-99^	-2.9368
MUGEST2003_23lib_Contig4148_13	EF368360	*Hordeum vulgare* WRKY transcription factor 7	5.41E^-80^	-2.3126

### Expression analysis of defense- and flowering-related genes

**Fig 4:**
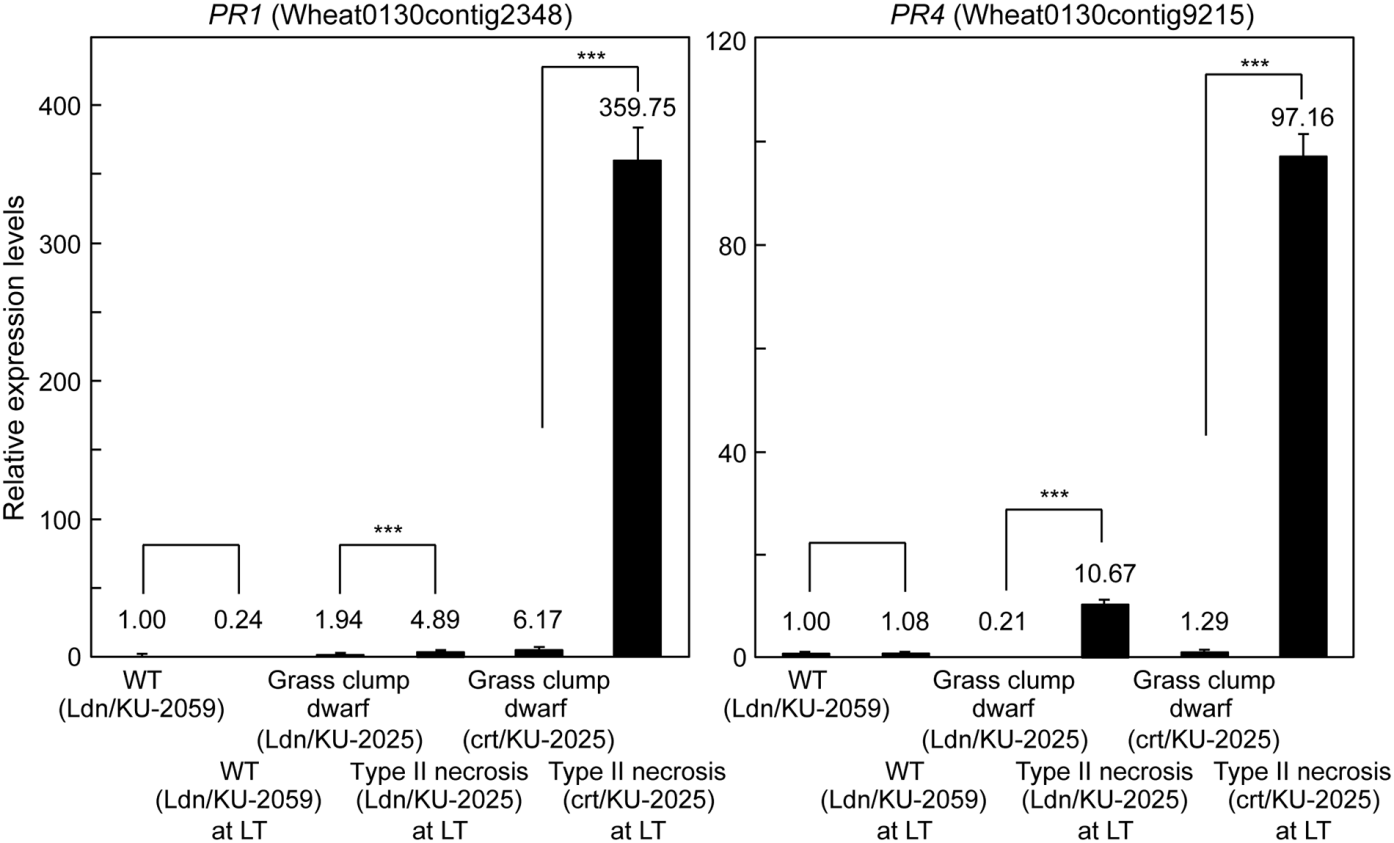
Comparison of transcript accumulation of the two defense-related genes in crown tissues of the WT and type II necrosis/grass-clump dwarf lines. The transcript accumulation levels were analyzed by qRT-PCR. The transcript levels are shown as values relative to those in crown tissues of Ldn/KU-2059 under normal temperature. Means ± SD were calculated from data obtained in three experiments. The *Actin* gene was used as an internal control. Student’s *t-*test was used to test for statistical significance (***P<0.001) between WT and type II necrosis/grass-clump dwarf synthetic plants.

The autoimmune response is triggered by interaction of the causal genes for hybrid necrosis. In most types of wheat hybrid necrosis, defense-related genes are dramatically up-regulated under both normal temperature and LT [14,17]. To validate the microarray data in the type II necrosis lines, qRT-PCR was conducted for two defense-related genes, *PR1* and *PR4*. In the crown tissues of WT synthetic wheat, no significant increases in transcript accumulation of these two defense-related genes were observed under LT (Fig 4). These defense-related genes were significantly up-regulated in the crown tissues of Ldn/KU-2025 and crt/KU-2025 under LT but not under normal temperature (Fig 4).

**Fig 5:**
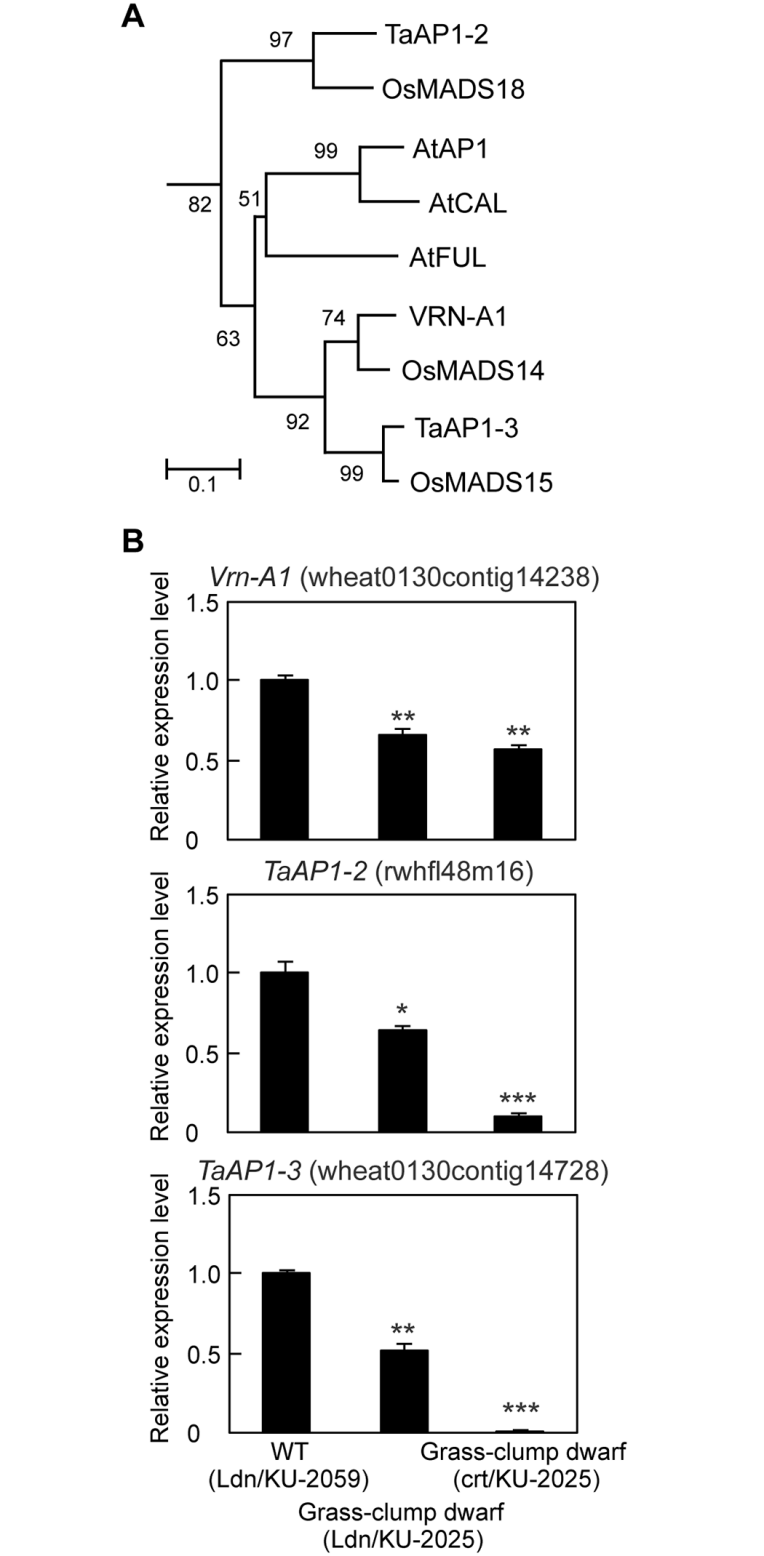
Comparison of transcript accumulation of the three MADS-box genes in crown tissues of the WT and type II necrosis/grass-clump dwarf lines. (A) Phylogenetic tree of the deduced MADS-domain amino acid sequences in the *AP1*/*FUL*-type MADS-box genes from *Arabidopsis*, rice and wheat. Bootstrap values after 1,000 replicates are shown at nodes. (B) Transcript accumulation levels of the three MADS-box gene transcripts from qRT-PCR. The transcript levels are shown as values relative to those in crown tissues of Ldn/KU-2059 under normal temperature. Means ± SD were calculated from data obtained in three experiments. The *Actin* gene was used as an internal control. Student’s *t-*test was used to test for statistical significance (***P<0.001) between WT and grass-clump dwarf synthetic plants.

All synthetic wheat lines used in this study contain a dominant *Vrn-A1* allele for spring habit from Ldn [54,55]. *Vrn-1*, a homolog of *Arabidopsis APETALA1* (*AP1*)/*FRUITFULL* (*FUL*), controls the vernalization requirement and acts as a flowering promoter [56,57]. Under normal temperature, many MADS-box genes were significantly down-regulated in the crown tissues of Ldn/KU-2025; the *Vrn-A1*-derived probe (wheat0130contig14238) showed 0.064 of the signal ratio relative to Ldn/KU-2059 in the microarray analysis. The signal ratios of two wheat *Vrn-1* homologs, *TaAP1-2* (rwhfl48m16) and *TaAP1-3* (wheat0130contig14728), were respectively 0.011 and 0.408 in the crown tissues of Ldn/KU-2025. Their rice orthologs *OsMADS18* and *OsMADS15* play important roles in the promotion of flowering [58,59], and phylogenetic relationship among the MADS domain-containing proteins in Fig 5A. To validate the microarray data, qRT-PCR was conducted for the three wheat *AP1*/*FUL*-like MADS-box genes. Transcript accumulation of the MADS-box genes acting as flowering promoters was markedly decreased in the crown tissues of Ldn/KU-2025 and crt/KU-2025 compared with Ldn/KU-2059 (Fig 5B).

### Alteration of expression profiles of miRNAs in type II necrosis lines of synthetic wheat

**Fig 6:**
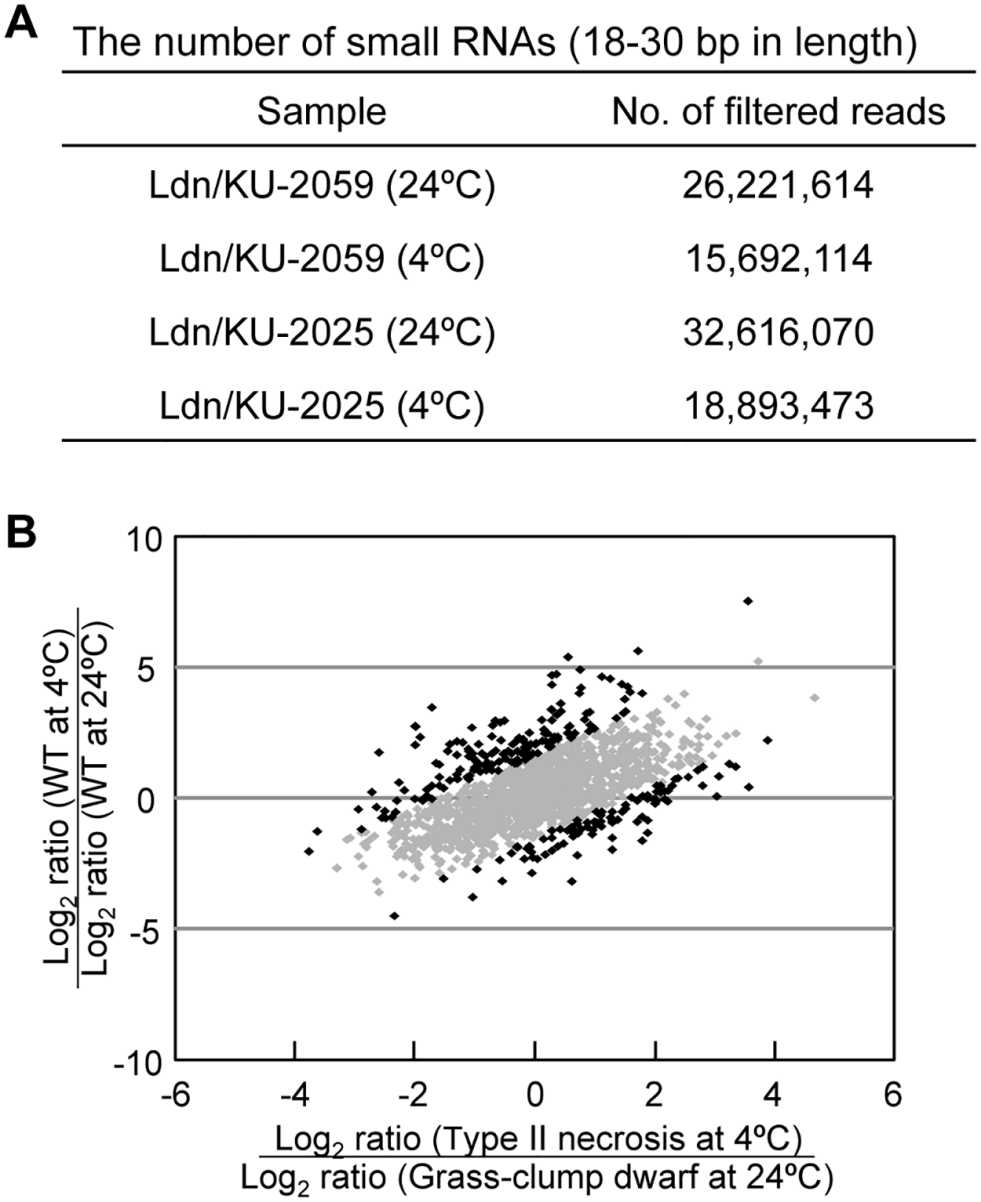
Differentially expressed miRNAs in crown tissues between the WT and type II necrosis/grass-clump dwarf lines. (A) Number of small RNAs from 18 bp to 30 bp in each sample used to identify known and novel miRNAs. (B) Scatter plot of the 1,620 miRNAs expressed in all samples. The *x*-axis represents the ratio of miRNAs expressed at 4°C and 24°C in Ldn/KU-2025 and the *y*-axis represents the ratio in Ldn/KU-2059. miRNAs whose ratio between Ldn/KU-2059 and Ldn/KU-2025 was ≥ 2 or ≤ 0.5 are indicated by dark dots.

Expression levels and patterns of small RNAs can be altered in interspecific hybrids and allopolyploids [30]. Moreover, many stress-responsive miRNAs were recently reported in higher plants including in wheat and relatives [23–26,60,61]. Therefore, we performed deep sequencing of small RNAs derived from four types of crown tissues for the first screening of miRNA associated with the grass-clump dwarfism: two from Ldn/KU-2025 and Ldn/KU-2059 grown under normal temperature (24°C) and two grown under LT (4°C). After trimming the adaptor sequence and filtering based on the nucleotide length (S1 Fig), more than 15 million small RNAs 18 to 30 bp in length were detected in each sample (Fig 6A). Repeat sequence-derived small RNAs and other non-coding RNAs (such as rRNA, tRNA, and snoRNAs) were removed, and then putative miRNAs were extracted based on alignment of reads to genomic sequences and prediction of miRNA loci. Known miRNAs were extracted from the putative miRNAs and remaining small RNAs using the BLASTn algorithm. Of the query sequences, small RNAs having ≥ 90% identity with the known miRNAs in miRbase were searched. In total, 48,695 miRNA sequences were obtained from the four types of crown tissues of the synthetic hexaploids, of which 6,514 corresponded to known miRNAs (S1 Fig and S4 Table). Of the 48,695 miRNAs, 1,620 were expressed in all samples.

To search for miRNAs differentially expressed between the WT and type II necrosis lines, we compared the log_2_ ratios of the expression levels of identified miRNAs under LT relative to those under normal temperature. Of the miRNAs, 1,357 (84.7%) showed similar ratios of expression under LT to normal temperature for Ldn/KU-2025 and Ldn/KU-2059 (Fig 6B). We screened miRNAs with ratios > 3.0 and < 1/3. In total, 245 small RNAs were selected as miRNAs differentially expressed in the crown tissues between the WT and type II necrosis lines. miR396, miR5054, miR156 and miR5072 showed lower responsiveness to the growth temperature in Ldn/KU-2059 than in Ldn/KU-2025 (Table 6). For miR1128, miR1135 and miR1136, higher responses to the growth temperature were observed in Ldn/KU-2059 than in Ldn/KU-2025. For miR159, miR166 and miR5048, both higher and less temperature-responsive molecules were found in Ldn/KU-2025.

**Table 6:** Top 15 differentially responsive miRNAs to growth temperature between crown tissues of WT and type II necrosis lines.

miRNA	Number of miRNA molecules	> 3.0 ratio of Ldn/KU-2059 to Ldn/KU-2025	< 1/3 ratio of Ldn/KU-2059 to Ldn/KU-2025	Putative targets
miR396	15	0	15	Growth Regulating Factor transcription factors
miR159	14	4	10	MYB Transcription factors
miR166	14	7	7	HD-Zip Transcription factors
miR5054	13	0	13	Pentatricopeptide repeat-containing protein At5g61400-like
miR1135	12	11	1	RNA recognition motif-containing protein-like, FAD-linked oxidoreductase, GRF zinc finger family, Delta1-pyrroline-5-carboxylate synthetase, HEAT repeat family
miR5048	10	6	4	Cysteine-rich receptor-like protein kinase
miR156	8	0	8	*SPL*s
miR169	8	3	5	CCAAT-binding transcription factor
miR168	7	0	7	Not found
miR6300	7	1	6	ras-related protein RABH1b-like
miR5072	6	0	6	Not found
miR1117	5	1	4	Non-specific lipid-transfer protein, receptor-like protein kinase FERONIA
miR1136	5	4	1	glycosyltransferase-like, Disease resistance protein RPM1, mitochondrial-processing peptidase subunit alpha-like
miR2916	5	2	3	18S small subunit ribosomal RNA gene, RRNA intron-encoded homing endonuclease
miR1128	4	4	0	Transcription factor-like protein DPB, glutamyl tRNA reductase, carbohydrate esterase, protein REVEILLE 6-like, CTD small phosphatase-like

**Fig 7:**
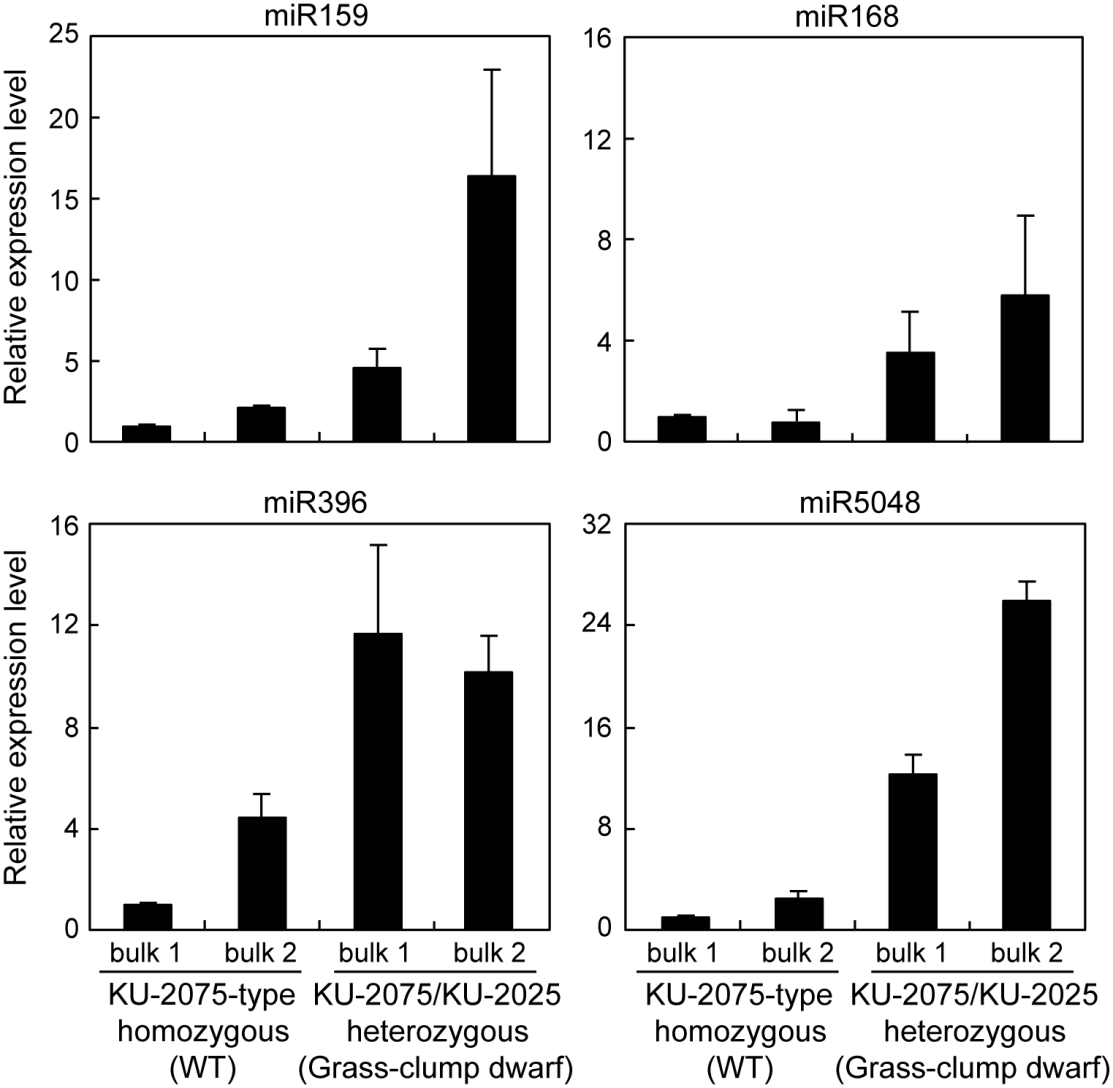
Comparison of transcript accumulation of the four wheat miRNAs in the KU-2075-type homozygous bulks and KU-2075/KU-2025 heterozygous bulks under normal temperature. Total RNA extracted from crown tissues of each genotype was bulked for qRT-PCR. Each bulked RNA sample was extracted from the crown tissues of five F_2_ individuals. The miRNA levels are shown as values relative to those in a KU-2075-type homozygous bulk. Means ± SD were calculated from data obtained in three experiments. 18S rRNA was used as an internal control.

To confirm the effect of the *Net2* genotype on miRNA accumulation, four miRNAs, miR159, miR168, miR396 and miR5048, were examined using two independently bulked crown tissues of each genotype, a KU-2075-allele homozygote (*Net2* non-carrier) and a KU-2075/KU-2025 heterozygote at the *Net2* chromosomal region. These were predicted to have differentially expressed miRNAs in the crown tissues between Ldn/KU-2059 and Ldn/KU-2025 (Table 6). Each bulked RNA sample was extracted from the crown tissues of five F_2_ individuals in the Ldn/KU-2075 and Ldn/KU-2025 mapping population grown in the experimental field of Kobe University on May (warm temperatures). Two samples of the heterozygous bulks more abundantly accumulated the four miRNAs than samples of the KU-2075-allele homozygous bulks (Fig 7). Thus, the KU-2025-derived *Net2* allele increased the accumulation of the four miRNAs in the crown tissues of synthetic wheat hexaploids under warm temperatures.

### Expression analysis of miR156 and *SPL* genes

**Fig 8:**
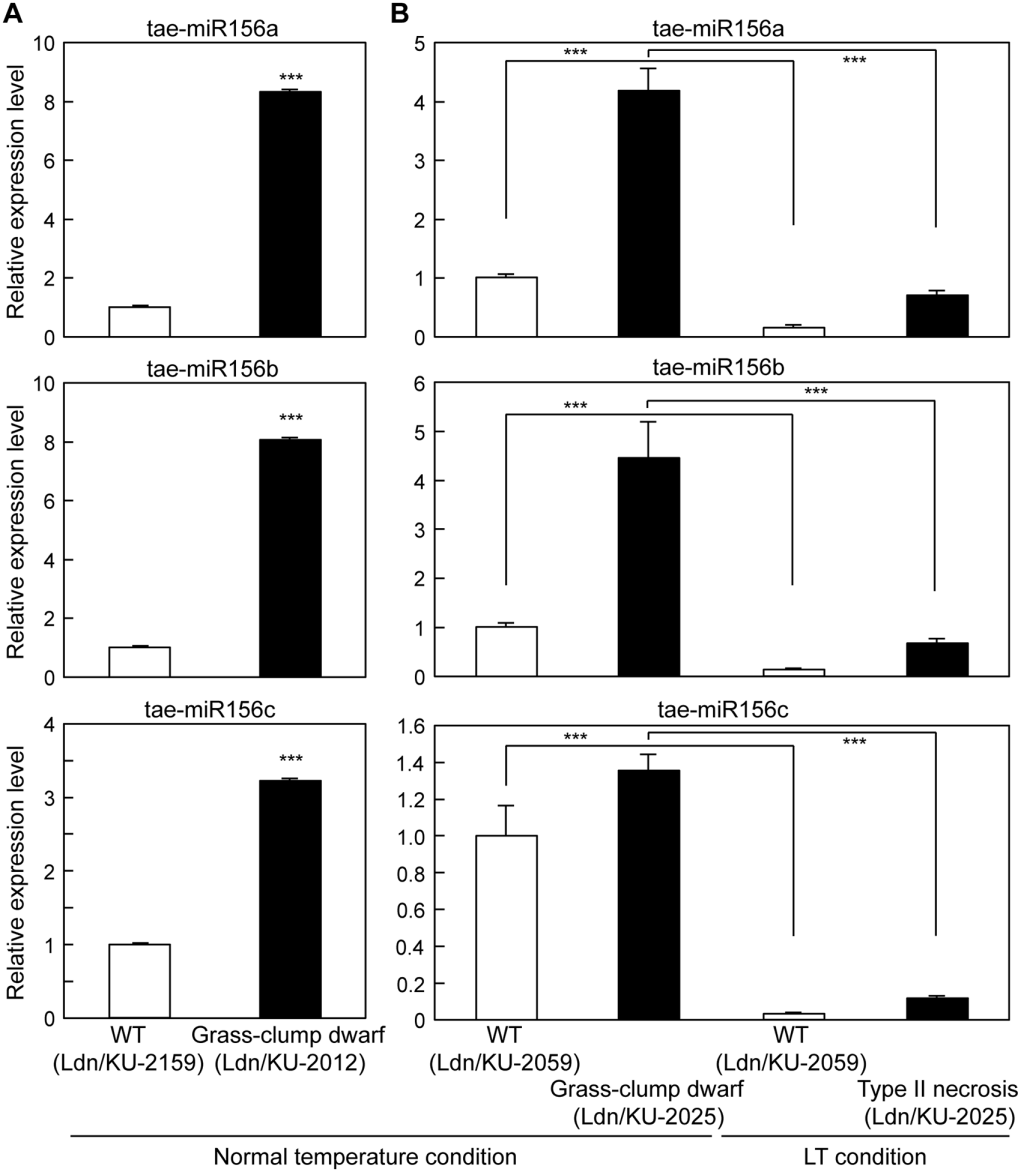
Comparison of transcript accumulation of the three miR156 molecules in the WT and type II necrosis/grass-clump dwarf lines. Means ± SD were calculated from data obtained in three qRT-PCR experiments. 18S rRNA was used as an internal control. (A) The miR156 levels in Ldn/KU-2159 and Ldn/KU2012 under normal temperature. Student’s *t*-test was used to test for statistical significance (***P<0.001) between crown tissues of the two lines under normal temperature. (B) The miR156 levels in crown tissues of Ldn/KU-2059 and Ldn/KU-2025. Student’s *t*-test was used to test for statistical significance (***P<0.001) in the two lines between growth at the normal temperature and LT.

In common wheat, several miR156 molecules have been previously identified, and their sequences are well conserved with those of other plant species [62]. At least three of the wheat miR156 molecules, tae-miR156a, tae-miR156b and tae-miR156c, showed significantly more abundant accumulation in the crown tissues of Ldn/KU-2012, a type II necrosis line, compared with Ldn/KU-2159, a WT line, under normal temperature (Fig 8A). A similar difference in the miR156 levels was confirmed between Ldn/KU-2059 and Ldn/KU-2025 under normal temperature, and the accumulation of the miR156 molecules was dramatically repressed by LT treatment in the crown tissues of both WT and type II necrosis lines (Fig 8B).

**Fig 9:**
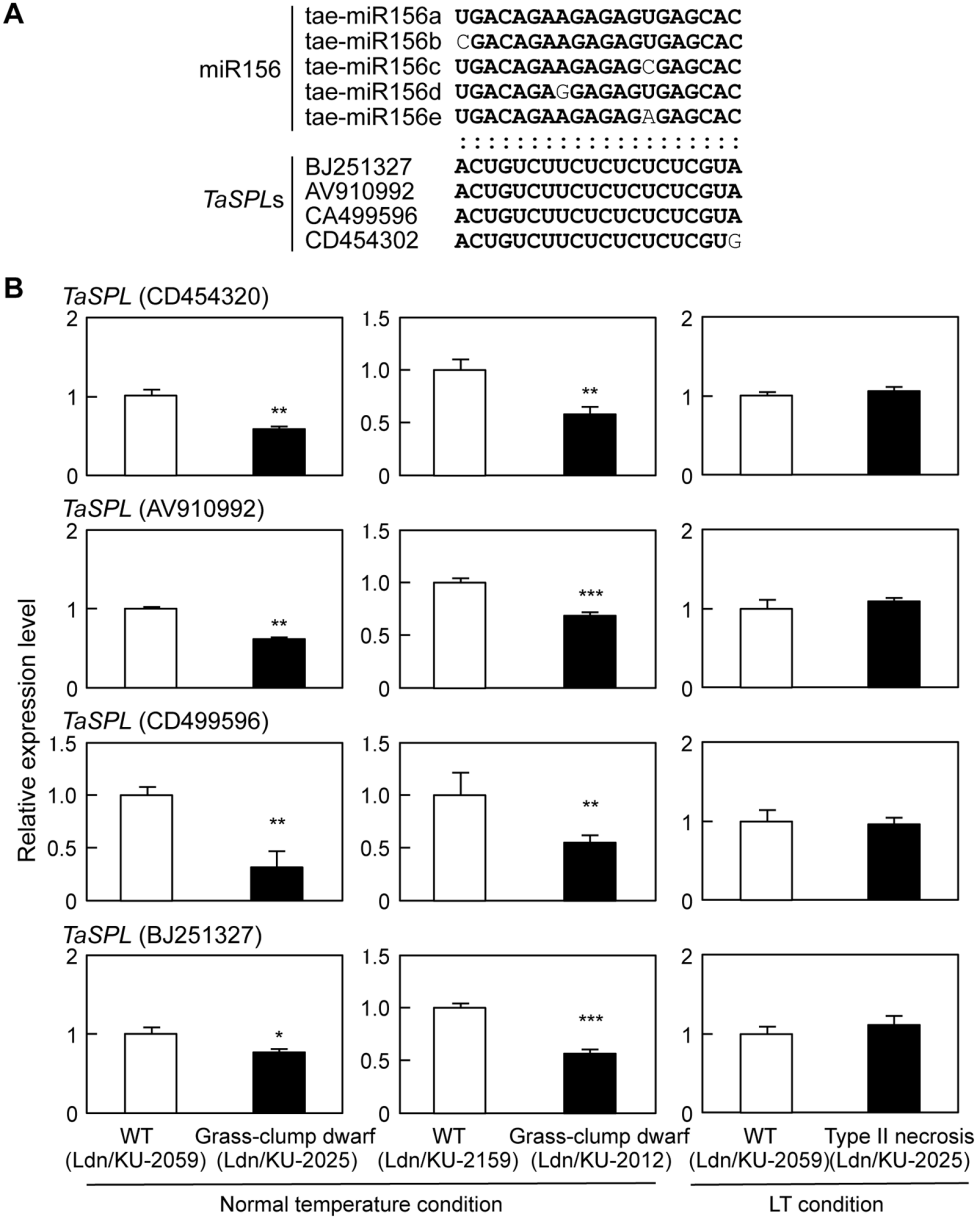
Expression analysis of the miR156-targeted wheat *SPL*s. (A) Complementary relationship between wheat miR156 molecules and their putative target sequences in *TaSPL*s. The reverse complement of five tae-miR156 targeting sites to the four *TaSPL* sequences is represented in bold letters. (B) Comparison of transcript accumulation levels of the four *TaSPL*s in crown tissues of the WT and type II necrosis/grass-clump dwarf lines by qRT-PCR. The transcript levels are shown as values relative to those in crown tissues of the WT line for each comparison. Means ± SD were calculated from data obtained in three experiments. The *Actin* gene was used as an internal control. Student’s *t*-test was used to test for statistical significance (*P<0.05, **P<0.01, ***P<0.001) between WT and type II necrosis/grass-clump dwarf synthetic plants.

*SPL* genes are associated with regulation of tillering and branching through interaction with miR156 in maize, rice and *Arabidopsis* [29,50,63]. In addition to these plant species, cleavage of an *SPL* mRNA via miR156 was previously confirmed in common wheat [64,65]. Our microarray studies showed that three wheat *SPL* genes (rwhoh17n20, MUGEST2003_23lib_Contig14620_504 and wheat0130Contig8035) were down-regulated in crown tissues of crt/KU-2025 under the normal temperature condition (S3 Table). We surveyed wheat *SPL* (*TaSPL*) cDNAs, and the putative amino acid sequences of TaSPLs were compared with those of *Arabidopsis* and rice SPLs (S3 Fig). Based on reports in which interaction between miR156 and *SPL*s of *Arabidopsis* and rice were confirmed [50,63], four *TaSPL*s were selected; the target sites of the five tae-miR156 molecules were present in the coding regions of the four *TaSPL*s (Fig 9A). Under normal temperature, the transcript accumulation levels of the four *TaSPL*s were significantly lower in the crown tissues of Ldn/KU-2025 than in Ldn/KU-2059 and in Ldn/KU-2012 than in Ldn/KU-2159 (Fig 9B). Under LT, however, no differences in transcript levels were observed in the four *TaSPL*s. To examine the cleavage sites of the *TaSPL* (CD454320) mRNA, 5’-RACE PCR with a *TaSPL-*specific primer was performed using the crown tissue-derived RNA of Ldn/KU-2059. One of six 5’-ends of the *TaSPL* mRNA was found in the target region of tae-miR156 molecules.

## Discussion

Type II necrosis is one of the major hybrid incompatibilities between tetraploid wheat and *Ae*. *tauschii*, and 22 of our 122 *Ae*. *tauschii* accessions display the type II necrosis phenotype in ABD triploids in the winter season [14]. As previously reported [16], necrotic symptoms appear in the type II necrosis lines of synthetic hexaploids only under LT, and elongation of stems and expansion of new leaves are repressed in the type II necrosis-displaying plants. When the growth temperature increases after the winter season, the type II necrosis lines then show a distinct phenotype of excess tillers and severely dwarfed culms [16,18]. Therefore, the phenotypic plasticity is growth temperature-dependent, and the temperature dramatically changes the growth phenotype from type II necrosis to grass-clump dwarf. The grass-clump dwarf phenotype of the type II necrosis lines shows the following four characteristics: no necrotic symptoms, excess tillers, severe dwarfism and delayed flowering. In these type II necrosis lines under normal temperature, the transcription levels of defense-related genes, which are up-regulated under LT, were significantly low (Fig 4). Thus, the LT-responsiveness of defense-related gene expression appears to be partly associated with the absence of a necrosis phenotype in the type II necrosis lines under normal temperature. In addition, significant repression of the wheat *AP1*/*FUL*-type MADS-box genes, the orthologs of which act as flowering promoters in rice [58,59], was observed at normal temperature (Fig 5). This repression could contribute to the delayed-flowering phenotype in the type II necrosis lines under normal temperature. These observations suggest that the transcriptional alterations of the defense-related genes and *AP1*/*FUL*-type MADS-box genes could explain at least partly two of the characteristics of the temperature-dependent phenotypic plasticity in the type II necrosis lines.

Information on molecular mechanisms underlying the other two characteristics of the grass-clump dwarf phenotype was obtained from deep-sequencing analysis of small RNAs. Accumulation of miR156 molecules was altered in response to the growth temperature in the crown tissues of wheat synthetic hexaploids (Fig 8). LT treatment dramatically inhibited miR156 accumulation in the crown tissues, and the miR156 molecules were more abundantly accumulated in the grass-clump dwarf lines than in WT lines under normal temperature. On the other hand, the transcript levels of *SPL*s, direct targets of miR156 [29,50,66], were significantly decreased in crown tissues of the grass-clump dwarf lines. Recent reports showed that wheat miR156 cleaves a *TaSPL* mRNA [64,65]. In addition, miR156 overexpression results in down-regulation of *SPL*s and in morphological changes including dwarfism, increased tiller number and late flowering in maize, switchgrass and rice [29,66,67], which have phenotypes that closely resemble the phenotype of the grass-clump dwarf lines [18]. The present study showed that at least four *TaSPL*s could be direct targets of miR156 in the crown tissues of wheat. Thus, the excess tiller numbers and dwarfism could be caused by the increased miR156 expression and repressed *TaSPL* expression in the grass-clump dwarf lines. Moreover, the miR156/*SPL*s module also controls flowering time in *Arabidopsis*. miR156 molecules decrease the expression of miR172 through the cleavage of *SPL* transcripts, and miR172 directly down-regulates *APETALA2*-like genes *TOE1* and *TOE2* to promote the transition from vegetative to reproductive phase [68]. Therefore, the increase in miR156 expression might induce lower miR172 levels, whereas no significant change in the miR172 levels in response to the growth temperature was observed in the crown tissues of the grass-clump dwarf lines. On the other hand, *Arabidopsis SPL*s directly activate flowering promoting the MADS-box genes *FUL* and *SOC1*, and miR156 molecules negatively regulate the MADS-box genes through the cleavage of *SPL* transcripts [69]. Therefore, the down-regulation of wheat *AP1*/*FUL*-type MADS-box genes could be due to the increased miR156 expression.

Evolution of allopolyploids is accompanied by changes in genome organization and gene expression patterns [2]. As recently reported, expression levels of a lot of genes are significantly distinct from the midparent values, and many of the non-additively expressed genes are likely to be underexpressed in nascent synthetic hexaploids exhibiting normal growth [70]. Expression levels and patterns of small RNAs could be altered in the interspecific hybrids and allopolyploids with normal growth phenotype [30,35]. In the present study, microarray-based transcriptome analysis and miRNA profiling showed that transcripts of a number of protein-coding genes and miRNA molecules were differentially accumulated in lines showing abnormal growth compared with WT lines of synthetic hexaploids (Figs 1 and 6). Most of the genes and small RNAs showing altered expression levels might have no relation with the growth abnormalities, and limited genes and small RNAs may visibly affect plant growth and development. For the grass-clump dwarf phenotype, unusual expression of only the miR156/*SPL*s module could at least partly explain the three characteristics of dwarfism, excess tillers and late flowering under normal temperature.

Recently, association of miRNA networks with developmental plasticity in higher plants has been discussed [71]. Some miRNAs including miR156, miR159 miR169 and miR319 are associated with coordination of the relationship between development and stress responses [61]. Our observations here show that the miR156/*SPL*s module is at least partly associated with the grass-clump dwarf phenotype in the type II necrosis lines of synthetic hexaploid wheat. Namely, the *Net1*-*Net2* interaction regulates the temperature-dependent phenotypic plasticity through the miR156/*SPL*s module in the wheat crown tissues. With *Net1*-*Net2* interaction, accumulation of some wheat miRNAs including miR156 showed unexpected alterations compared with the WT lines in the wheat ABD hybrids and synthetic hexaploids (Fig 6 and Table 6). Modified expression of miRNAs has been reported during the allopolyploidization process of *A*. *suecica* and in interspecific ABD hybrids and newly synthesized wheat hexaploids [30,31,35,36]. During interspecific hybridization and the allopolyploidization that follows, miRNA expression is largely altered, and some of these alterations could negatively affect plant growth and development. The growth abnormalities related to the modified miRNA levels could reduce the fitness of the hybrids and allopolyploids and function as postzygotic hybridization barriers between different nuclear genomes.

Under LT, the *Net1*-*Net2* interaction triggers an autoimmune response-like reaction followed by appearance of necrotic symptoms in wheat ABD hybrids and synthetic hexaploids [16]. Disease resistance-related genes have been identified as causal genes for hybrid necrosis, and the interaction explained by the DM model plays important roles in differentiation and establishment of new genealogical lineages in plants [1,4]. In the case of *Net1*-*Net2* interaction, however, critical responses for phenotypic plasticity might occur in the crown tissues. Necrotic cell death is usually observed in leaves, whereas arrest of cell division at the shoot apical meristem seems to occur prior to the autoimmune response in type II necrosis lines as well as in the severe growth abortion of wheat ABD hybrids [16,38]. In the grass-clump dwarf, modification of the miR156/*SPL*s module occurs at the shoot apical meristem of the crown tissues under normal temperature. Therefore, the cell death induced by the autoimmune response might be a secondary event in type II necrosis. In fact, suppression of cell cycle-related gene expression induces both growth inhibition and necrotic cell death in *Arabidopsis* [72,73]. Therefore, dramatic alteration of gene expression profiles, including miRNA levels, at the shoot apical meristem induced by a DM gene interaction could be strongly associated with the growth abnormalities in wheat ABD hybrids and synthetic hexaploids.

Here, we showed comparative transcriptome analyses of the crown tissues from growth temperature-dependent phenotypic abnormalities-showing wheat hybrids. The transcriptome analyses clearly revealed that dramatic alteration of gene expression profiles, including miRNA levels, in crown tissues was associated with the phenotypic plasticity in type II necrosis/grass-clump dwarf wheat hybrids. Especially, unusual expression patterns of the miR156/*SPL*s module could well explain the grass-clump dwarf phenotype. The type II necrosis/grass-clump dwarfism is induced by the *Net1*-*Net2* complementary gene interaction. Thus, the *Net1*-*Net2* interaction appears to control the miR156/*SPL*s module in response to the growth temperature in the wheat ABD hybrids and synthetic hexaploids. The molecular mechanisms controlling the miR156/*SPL*s module regulation via the *Net1*-*Net2* interaction are unknown. The *Net1*-*Net2* interaction induces the growth abnormalities: necrotic cell death triggered by an autoimmune response under LT and the grass-clump dwarf phenotype induced through the miR156/*SPL*s module under normal temperature. The DM interaction is greatly affected by the growth temperature, and thus the third component, connecting the *Net1*-*Net2* interaction to its downstream targets, must be a key factor for the temperature-dependent phenotypic plasticity. Molecular cloning of the causal genes, *Net1* and *Net2*, should be the next aim of study to clarify the molecular nature of phenotypic plasticity in the wheat ABD hybrids and synthetic hexaploids.

## Supporting information

S1 Fig Identification of microRNAs in crown tissues of WT and type II necrosis lines. First, adapter sequences were trimmed from raw reads and those containing a stop oligonucleotide were discarded. The trimmed reads were BLASTn searched against the Rfam database to remove sequences derived from non-coding RNAs other than miRNAs. Then reads ≥ 18 bp and ≤ 30 bp were selected and aligned to repeat-masked A- and D-genome sequences to search for putative miRNA loci. BLASTn searches against the miRBase were performed to distinguish known miRNAs from novel ones. The same BLAST search was performed with reads unaligned to the genome that remained after Mireap analysis to extract known miRNAs.
(PDF)

S2 Fig Correlation of the log_2_ ratios of the genes with expression altered between WT (Ldn/KU2059) and two grass-clump dwarf (Ldn/KU2025 and crt/KU2025) lines. Scatter plots of differential signal intensities in crown tissues of the grass-clump dwarf lines at the normal temperature are represented for the six indicated categories of probes. The correlations were significant (***P<0.001). The regression lines are also shown.
(PDF)

S3 Fig Phylogenetic tree of the SPL family based on the amino acid sequences of SBP domains. Recent reports have shown [51,63] that the underlined *SPL* genes contain the miR156-target site, and their transcripts are directly cleaved by miR156 in *Arabidopsis* and rice.
(PDF)

S1 Table Primer sets used for mRNA qRT-PCR analysis. (PDF)

S2 Table Primers used for miRNA qRT-PCR analysis. (PDF)

S3 Table Gene probes identified by microarray analysis as up- and down-regulated in crown tissues. (XLSX)

S4 Table List of miRNAs expressed in crown tissues of the WT and type II necrosis lines. (XLSX)
