# Supplementary material for: Global gene expression profiling related to temperature-sensitive growth abnormalities in interspecific crosses between tetraploid wheat and Aegilops tauschii
Source: PLoS One. 2017 May 2;12(5):e0176497. doi: 10.1371/journal.pone.0176497 (PMC5413045; doi:10.1371/journal.pone.0176497)
Supplement: S1 Fig — First, adapter sequences were trimmed from raw reads and those containing a stop oligonucleotide were discarded. The trimmed reads were BLASTn searched against the Rfam database to remove sequences derived from non-coding RNAs other than miRNAs. Then reads ≥ 18 bp and ≤ 30 bp were selected and aligned to repeat-masked A- and D-genome sequences to search for putative miRNA loci. BLASTn searches against the miRBase were performed to distinguish known miRNAs from novel ones. The same BLAST search was performed with reads unaligned to the genome that remained after Mireap analysis to extract known miRNAs. (PDF) [file pone.0176497.s001.pdf]

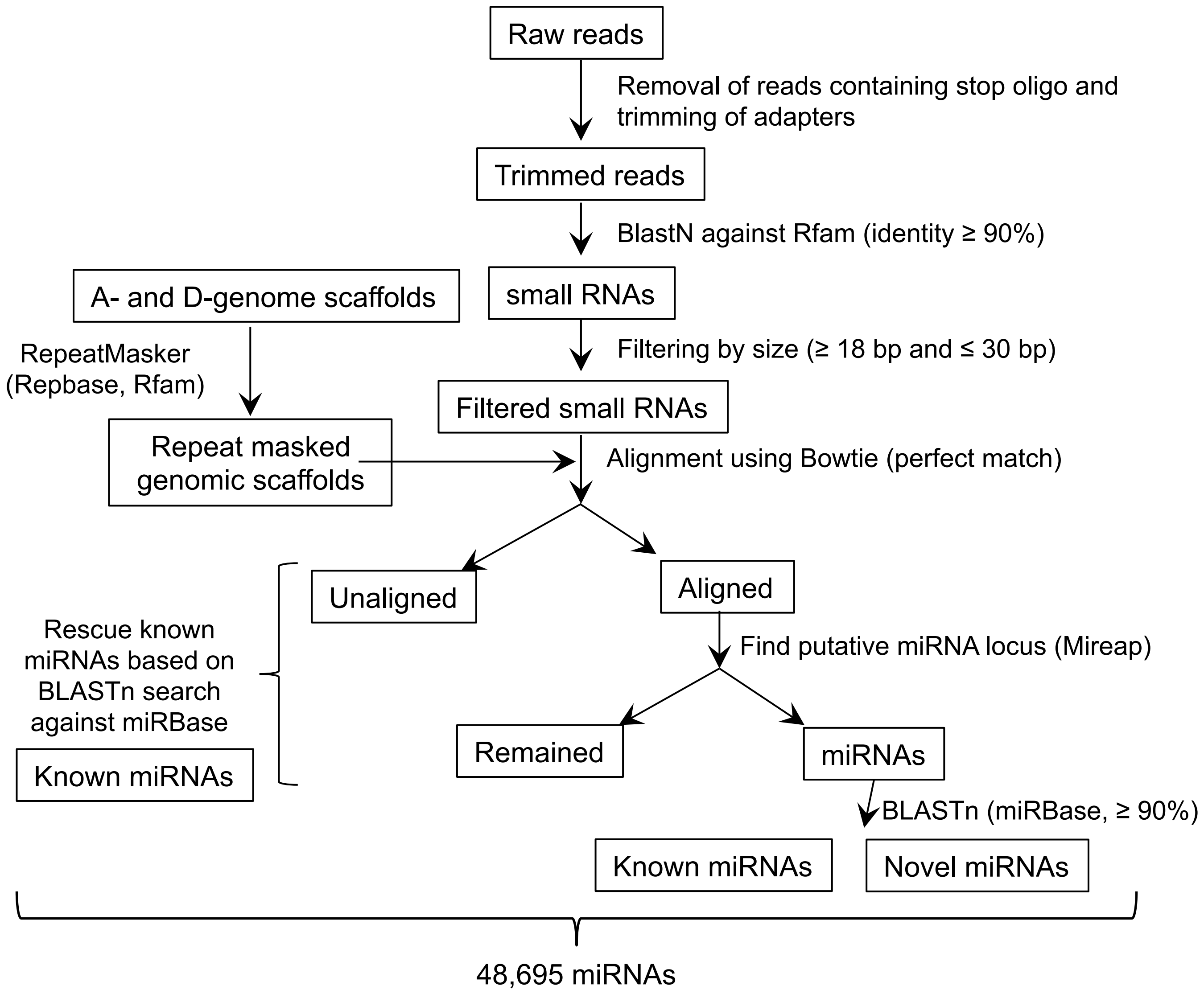

S1 Fig. Identification of microRNAs in crown tissues of WT and type II necrosis lines. First, adapter sequences were trimmed from raw reads and those containing a stop oligonucleotide were discarded. The trimmed reads were BLASTn searched against the Rfam database to remove sequences derived from non-coding RNAs other than miRNAs. Then reads ≥ 18 bp and ≤ 30 bp were selected and aligned to repeat-masked A- and D-genome sequences to search for putative miRNA loci. BLASTn searches against the miRBase were performed to distinguish known miRNAs from novel ones. The same BLAST search was performed with reads unaligned to the genome that remained after Mireap analysis to extract known miRNAs.
