# Supplementary material for: Global gene expression profiling related to temperature-sensitive growth abnormalities in interspecific crosses between tetraploid wheat and Aegilops tauschii
Source: PLoS One. 2017 May 2;12(5):e0176497. doi: 10.1371/journal.pone.0176497 (PMC5413045; doi:10.1371/journal.pone.0176497)
Supplement: S2 Fig — Scatter plots of differential signal intensities in crown tissues of the grass-clump dwarf lines at the normal temperature are represented for the six indicated categories of probes. The correlations were significant (***P<0.001). The regression lines are also shown. (PDF) [file pone.0176497.s002.pdf]

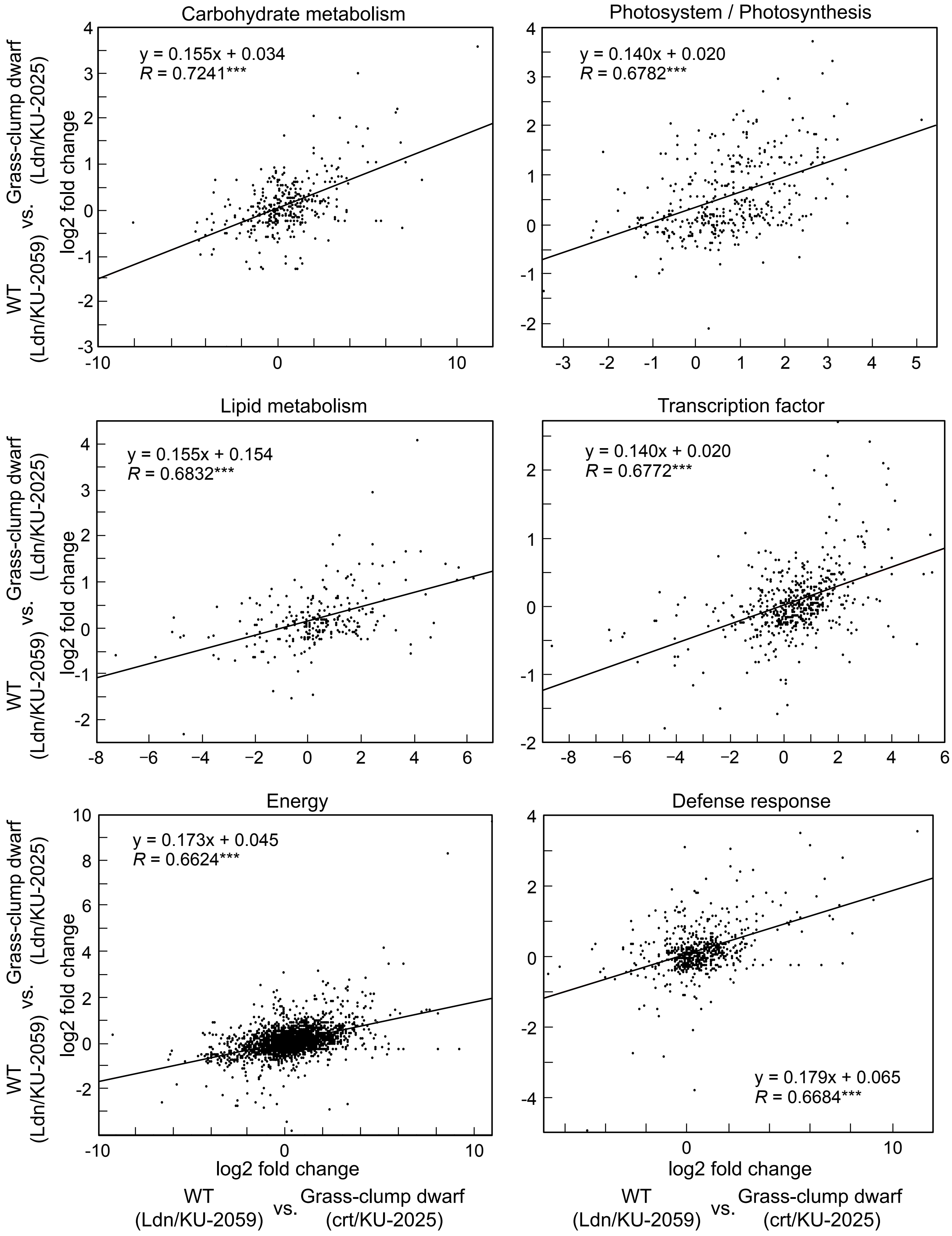

S2 Fig. Correlation of the $\log_2$ ratios of the genes with expression altered between WT (Ldn/KU2059) and two grass-clump dwarf (Ldn/KU2025 and crt/KU2025) lines. Scatter plots of differential signal intensities in crown tissues of the grass-clump dwarf lines at the normal temperature are represented for the six indicated categories of probes. The correlations were significant (****P*<0.001). The regression lines are also shown.
