# Supplementary material for: Global gene expression profiling related to temperature-sensitive growth abnormalities in interspecific crosses between tetraploid wheat and Aegilops tauschii
Source: PLoS One. 2017 May 2;12(5):e0176497. doi: 10.1371/journal.pone.0176497 (PMC5413045; doi:10.1371/journal.pone.0176497)
Supplement: S3 Fig — Recent reports have shown [51,63] that the underlined SPL genes contain the miR156-target site, and their transcripts are directly cleaved by miR156 in Arabidopsis and rice. (PDF) [file pone.0176497.s003.pdf]

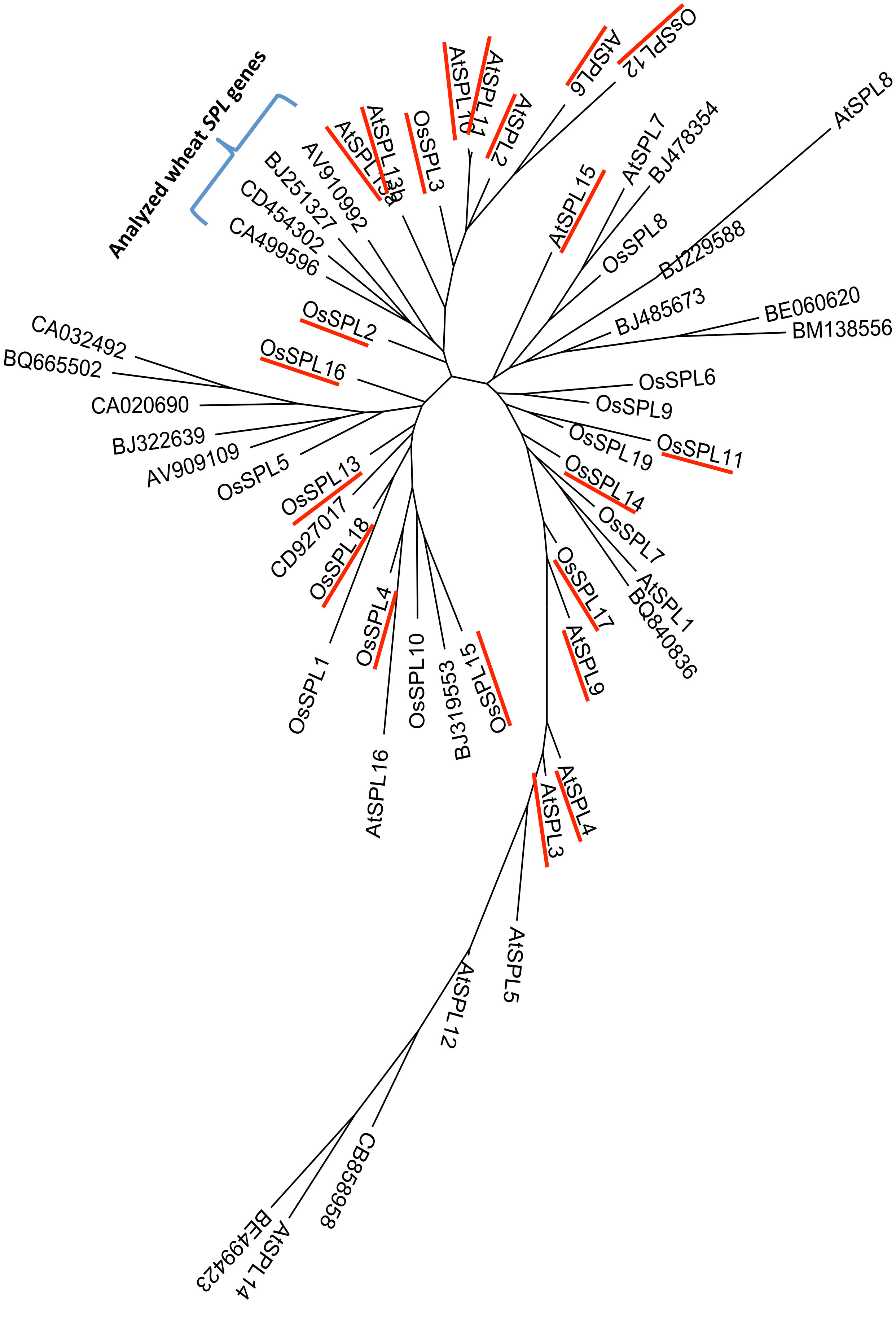

S3 Fig. Phylogenetic tree of the SPL family based on the amino acid sequences of SBP domains. Recent reports have shown [52,64] that the underlined *SPL* genes contain the miR156-target site, and their transcripts are directly cleaved by miR156 in *Arabidopsis* and rice.
