# Supplementary material for: Global gene expression profiling related to temperature-sensitive growth abnormalities in interspecific crosses between tetraploid wheat and Aegilops tauschii
Source: PLoS One. 2017 May 2;12(5):e0176497. doi: 10.1371/journal.pone.0176497 (PMC5413045; doi:10.1371/journal.pone.0176497)
Supplement: S1 Table — (PDF) [file pone.0176497.s004.pdf]

S1 Table. Primer sets used for mRNA qRT-PCR analysis.

Gene	Forward primer sequence (5'~3')	Reverse primer sequence (5'~3')
TaSPL (AV910992)	AGAGTGCATCTGGGGACTTG	ATCATGGTTTCGATGGGTTC
TaSPL (BJ251327)	CAGAGCCCCACCGATGAT	TACCCTTCCAGGTGTCCAAG
TaSPL (CD454320)	AGGCATTGCCAAACCATCCG	AGCTCCCAAAGCCTTCCTAC
TaSPL (CD499596)	TGGCTAGCACCAGTGAATTG	TGCTGTTCCAGCACTGAGTC
PR1	GCAGAACTCGCCTCAGGACT	GCCGAGGTTATTGTTGCAGA
PR4	CGCGGTGAGCGCCTACTG	CCGATCCCGTTGGTG
Vrn-A1 (wheat0130contig14238)	ATCAGACTCAGCCTCAAACA	TAGAGACGGGTATCATGGAA
TaAP1-2 (rwhfl48m16)	TGGAGCAACAACAAGCTCAC	CAGCCTTCATCTGTTGCTGA
TaAP1-3 (wheat0130contig14728)	ATCTACCACCGTGGATGCTC	CAACCACAAGATCGTGCTCA
Actin	GCCGTGCTTTCCCTCTATG	GCTTCTCCTTGATGTCCCTTA
