# Supplementary material for: Global gene expression profiling related to temperature-sensitive growth abnormalities in interspecific crosses between tetraploid wheat and Aegilops tauschii
Source: PLoS One. 2017 May 2;12(5):e0176497. doi: 10.1371/journal.pone.0176497 (PMC5413045; doi:10.1371/journal.pone.0176497)
Supplement: S2 Table — (PDF) [file pone.0176497.s005.pdf]

S2 Table. Primers used for miRNA qRT-PCR analysis.

miRNA	Primer sequence (5' – 3')
miR159	TTTGGATTGAAGGGAGCTCTA
miR168	TCGCTTGGTGCAGATCGGGAC
miR396	TCCACAGGCTTTCTTGAACTG
miR5048	TATGTTTGCAGGTTTTAGGTCT
tae-miR156a	TGACAGAAGAGAGTGAGCAC
tae-miR156b	CGACAGAAGAGAGTGAGCAC
tae-miR156c	TGACAGAAGAGAGCGAGCAC
18S rRNA	AGGCCTTCACCAAGTATGCTCTGA and TGGGCGATAACACGGACAACAGTA
